# Lovastatin inhibits erythroleukemia progression through KLF2-mediated suppression of MAPK/ERK signaling

**DOI:** 10.1186/s12885-023-10742-4

**Published:** 2023-04-04

**Authors:** Jian Gao, Jifen Hu, Fang Yu, Chunlin Wang, Danmei Sheng, Wuling Liu, Anling Hu, Kunling Yu, Xiao Xiao, Yi Kuang, Eldad Zacksenhaus, Babu Gajendran, Yaacov Ben-David

**Affiliations:** 1 State Key Laboratory for Functions and Applications of Medicinal Plants, Guizhou Medical University, Guiyang, Guizhou, 550014 People’s Republic of China; 2 The Key Laboratory of Chemistry for Natural Products of Guizhou Province and Chinese, Academic of Sciences, Guiyang, Guizhou People’s Republic of China; 3 Department of Medicine, University of Toronto, Toronto, ON Canada; 4 Division of Advanced Diagnostics, Toronto General Research Institute, University Health Network, Toronto, ON Canada; 5 School of Pharmaceutical Sciences, Guizhou Medical University, Guiyang, Guizhou Province 550025 People’s Republic of China

**Keywords:** Lovastatin, Leukemia progression, KLF2, FAM38A, ERK1/2, Cholesterol biosynthesis

## Abstract

**Background:**

Lovastatin, an HMG-CoA inhibitor and an effective cholesterol lowering drug, exhibits anti-neoplastic activity towards several types of cancer, although the underlying mechanism is still not fully understood. Herein, we investigated mechanism of growth inhibition of leukemic cells by lovastatin.

**Methods:**

RNAseq analysis was used to explore the effect of lovastatin on gene expression in leukemic cells. An animal model of leukemia was used to test the effect of this statin in vivo. FAM83A and DDIT4 expression was knocked-downed in leukemia cells via lentivirus-shRNA. Western blotting, RT-qPCR, cell cycle analysis and apoptosis assays were used to determine the effect of lovastatin-induced growth suppression in leukemic cells in vitro.

**Results:**

Lovastatin treatment strongly inhibited cancer progression in a mouse model of erythroleukemia induced by Friend virus. In tissue culture, lovastatin inhibited cell proliferation through induction of G_1_ phase cell cycle arrest and apoptosis. Interestingly, lovastatin induced most known genes associated with cholesterol biosynthesis in leukemic cells. Moreover, it suppressed ERK1/2 phosphorylation by downregulating FAM83A and DDIT4, two mediators of MAP-Kinase signaling. RNAseq analysis of lovastatin treated leukemic cells revealed a strong induction of the tumor suppressor gene KLF2. Accordingly, lentivirus-mediated knockdown of KLF2 antagonized leukemia cell suppression induced by lovastatin, associated with higher ERK1/2 phosphorylation compared to control. We further show that KLF2 induction by lovastatin is responsible for lower expression of the FAM83A and DDIT4 oncogenes, involved in the activation of ERK1/2. KLF2 activation by lovastatin also activated a subset of cholesterol biosynthesis genes that may further contribute to leukemia suppression.

**Conclusions:**

These results implicate KLF2-mediated FAM83A/DDIT4/MAPK suppression and activation of cholesterol biosynthesis as the mechanism of leukemia cell growth inhibition by lovastatin.

**Supplementary Information:**

The online version contains supplementary material available at 10.1186/s12885-023-10742-4.

## Introduction

Statins act as inhibitors of hydroxymethyl glutaryl coenzyme A reductase (HMG-CoA) to reduce blood cholesterol, and are commonly used to diminish the risk of cardiovascular diseases [1]. Lovastatin (Lova) was the first statin to be approved by the US FDA in 1987 as a cholesterol-lowering drug. It blocks the conversion of HMG-CoA to mevalonate by inhibiting the function of HMG-CoA reductase (HMGCR) enzyme [2]. In addition to reducing cholesterol activity, anti-cancer effects of lovastatin have been reported in some cancer types [3, 4], including breast cancer [5], ovarian cancer [6] and multiple myeloma [7]. However, the role of cholesterol in cancer progression is controversial and the mechanism of statins in this context are not yet fully established.

While many studies implicated high serum cholesterol in cancer initiation and progression [8–13], other studies found no association [14] or even tumor inhibition [15, 16]. Indeed, we previously identified limonoid compounds exhibiting potent anti-leukemia activity [17]. Mechanistically, we have shown that these compounds act as agonists of ERK1/2. Overt and uncontrol activation of the MAPK pathway induced by limonoid in leukemia cell lines strongly induces upregulation of cholesterol biosynthesis and other pathways, leading to a block in leukemia progression [18]. This is consistent with reports that MAPK is upstream of cholesterol biosynthesis [19]. Accordingly, inhibition of cholesterol pathway by statin partially prevented growth suppression induced by these limonoid compounds [18].

The family with sequence similarity 83 member A (FAM83A) oncogene is associated with the development of many malignant tumors [20–25]. FAM83A interacts with the RAS pathway to drive the activation of PI3K and MAPK signaling [20, 21]. High level of FAM83A expression maintains critical levels of MEK/ERK survival signaling and blocks cell death in pancreatic cancer cells [21]. FAM83A is thus proposed as a major driver of cancer progression through its direct interaction with MAPK signaling. DNA damage inducible transcript 4 (DDIT4), expressed under stress situation, also acts as a mediator of cell growth in cancer cells through inhibition of mTORC1 [26]. Similar to FAM83A, DDIT4 activates the MAPKinase pathway to accelerate gastric cell proliferation, although the underlying mechanism still unknown [27]. Both FAM83A and DDIT4 are potential target for drug development.

Although lovastatin lowers serum cholesterol, its effect on tumor cell cholesterol level is unclear. Since lovastatin is known to block MAPK, blocking this signaling pathway may be in part responsible for its tumor growth inhibition [19]. Here, we show that high doses of lovastatin while inhibiting MAPK signaling, surprisingly also induce high expression of genes associated with cholesterol biosynthesis. RNAseq analysis identified robust upregulation of the transcription factor KLF2 by lovastatin, previously known for its tumor suppressor activity [28]. We have shown for the first time that KLF2 upregulation by lovastatin causing downregulation of both growth promoting genes FAM83A and DDIT4, leading to suppression of its downstream MAPK/ERK pathway. Inhibition of MAPK/ERK and higher cholesterol activity by KLF2 are likely mechanisms by which lovastatin suppressed leukemia progression in culture and in vivo.

## Materials and methods

### Cells and culture conditions

The human erythroleukemia cell lines HEL and K562 was previously obtained from ATCC (HEL 92.1.7, K562 CLL-243), and maintained mycoplasma free, as previously described [29]. The generation of mouse erythroleukemia cell line CB3 was previously described [30]. HEK293T cells were originally obtained from ATCC (CRL-3216). Cells were cultured and maintained in Dulbecco’s Modified Eagle Medium supplemented with HyClone 5% fetal bovine serum (GE Healthcare).

Cells were treated with lovastatin with indicated concentrations/times and used for cell counting or MTT assay (to determine the proliferation index) and for protein/mRNA extraction (for western blotting and RT-qPCR). Lovastatin compound was purchased from Solarbio, China.

### shRNA expression

The shRNA lentiviruses used was constructed, as previously described [31]. Briefly, shKLF2 and scrambled control vectors were generated by inserting the KLF2 shRNA and scrambled DNAs into the restriction enzyme sites BcuI within the PLent-GFP expression vector (obtained from Vigene Bioscience, Rockville, MD, USA). The lentivirus particles were generated by co-transfecting shKLF2 DNAs (10 µg) with packaging plasmids psPAX2 (5 µg) and pMD2.G (10 µg) (Addgene plasmid #12,259 & #12,260) into HEK293T cells, using Lipofectamine 2000 [30, 31]. Similar strategy is used to generate shDDIT4 lentivirus. Two days after transfection, the supernatants were collected and used to transduce HEL cells [31]. The positive expressing cells were then selected after incubation with medium containing puromycin (2 µg/ml; Solarbio). The sequences of the shKLF2s and shDDIT4 are shown in Table 1.

**Table 1:** shKLF2 gene sequences

Gene	Sequence
shKLF2/1	5’CGGCACCGACGACGACCTCAATTCAAGAGATTGAGGTCGTCGT CGGTGCCGTTTTTT3’
shKLF2/2	5’AGTTCGCATCTGAAGGCGCATTTCAAGAGAATGCGCCTTCAGAT GCGAACTTTTTTT3’
shKLF2/3	5’CACCGGCCATTCCAGTGCCATTTCAAGAGAATGGCACTGGAATG GCCGGTGTTTTTT3’
shDDIT4/1	5’GATGCCTAGCCAGTTGGTAAGTTCAAGAGACTTACCAACTGGCT AGGCATCTTTTTT3’
shDDIT4/2	5’GTCAGTGACCCTGAGGATGAACTTCAAGAGAGTTCATCCTCAGG GTCACTGATTTTTT3’
shDDIT4/3	5’GCACTGGCTTCCGAGTCATCAATTCAAGAGATTGATGACTCGGAA GCCAGTGTTTTTT 3’

### RNA preparation and RT-qPCR

For RT-qPCR, total RNA is extracted using TRIzol reagent (Life Technologies; Thermo Fisher Scientific, USA). CDNA was synthesized by using the PrimeScript RT Reagent kit (Takara Bio, Beijin, China). RT-qPCR analysis was done using the FastStart Universal SYBR Green Master Mix (Roche, Shanghai, China) and the Step One Plus Real time PCR system (Applied Biosystems/Thermo Fisher Scientific, US). The expression of the test genes was given as relative values to the expression of GAPDH. Three biological replicates were used for all the RT-qPCRs, each in triplicate (n = 3). Primers sequence list in the Table 2.

### Western blotting

Western blotting was performed as previously described [30, 31]. The antibodies used are as follows: Polyclonal rabbit antibodies for FLI1 (ab133485), DDIT4 (AB191871) and total ERK (ab184699) were purchased from Abcam; KLF2 (#46,591) and GAPDH (G9545) antibodies were obtained from Sigma Aldrich; β actin (20,536 1 AP) and FAM83A (20618-1-AP) antibodies were obtained from Proto Technology (Proteintech, Bucuresti, Romania); Phospho-ERK (Thr202/Tyr204; Cat. no. 9101s), goat anti mouse and goat anti rabbit HRP conjugated antibodies were obtained from Cell Signaling Technology (Cat. no. 5470s and 5151s, respectively). Antibody dilution was conducted according to the manufacturer’s instructions. The Odyssey system (LI COR Biosciences) and Bio was used to image proteins in western blot analysis.

**Table 2:** RT-qPCR gene primers sequence

Gene	Forward	Reverse
GAPDH	GGAGCGAGATCCCTCCAAAAT	GGCTGTTGTCATACTTCTCATGG
KLF2	TTCGGTCTCTTCGACGACG	TGCGAACTCTTGGTGTAGGTC
APOA1	CCCTGGGATCGAGTGAAGGA	CTGGGACACATAGTCTCTGCC
FAM83A	GGCCCTAAGGGACTGGACT	CACAGTGGCGCTGGATTTTT
HMGCS1	GATGTGGGAATTGTTGCCCTT	ATTGTCTCTGTTCCAACTTCCAG
HMGCR	TGATTGACCTTTCCAGAGCAAG	CTAAAATTGCCATTCCACGAGC
MVK	CATGGCAAGGTAGCACTGG	GATACCAATGTTGGGTAAGCTGA
MVD	CTCCCTGAGCGTCACTCTG	GGTCCTCGGTGAAGTCCTTG
IDI1	AACACTAACCACCTCGACAAGC	AGACACTAAAAGCTCGATGCAA
FDPS	TGTGACCGGCAAAATTGGC	GCCCGTTGCAGACACTGAA
TM7SF2	GTCGCCTGCGCTATCCTATTA	TGCGCCTTCATGTAGAGAAAGA
DDIT4	TGAGGATGAACACTTGTGTGC	CCAACTGGCTAGGCATCAGC
FDFT1	CCACCCCGAAGAGTTCTACAA	TGCGACTGGTCTGATTGAGATA
DHCR7	GCTGCAAAATCGCAACCCAA	GCTCGCCAGTGAAAACCAGT
DHCR24	GCCGCTCTCGCTTATCTTCG	GTCTTGCTACCCTGCTCCTT
SC5D	ACCATACGTGTATCCAGCCAC	GCTCAGTGTTGCACAGAAGAAA
EBP	CTCAGCACCTAAGACTGGACA	ACGACTAAGACCCCTGTGACA
HSD17B7	GTGCTGGTGTGTAACGCAG	GTCCCTACTACATTCACGTCCA
MSMO1	TGCTTTGGTTGTGCAGTCATT	GGATGTGCATATTCAGCTTCCA
CYP51A	GAAACGCAGACAGTCTCAAGA	ACGCCCATCCTTGTATGTAGC
NSDHL	CAAGTCGCACGGACTCATTTG	ACTGTGCATCTCTTGGCCTG
SQLE	GGCATTGCCACTTTCACCTAT	GGCCTGAGAGAATATCCGAGAAG
LSS	GCACTGGACGGGTGATTATGG	TCTCTTCTCTGTATCCGGCTG

### RNAseq analysis

The RNAseq data presented in **Supplementary Table 1** was generated using RNA isolated from HEL cells treated with DMSO or 20 µM of lovastatin for 24 h, as previously described [31]. Differentially expressed genes (DEGs) with at least 2.5 higher or lower were identified and shown in **Supplementary Table 1**.

### Leukemia induction in mice and drug therapy

In our mouse model of erythroleukemia, one day old BALB/c mice were inoculated intraperitoneally (i.p.) with Friend Murine Leukemia Virus (F-MuLV), as previously described [30]. Five weeks post viral infection, mice were injected intraperitoneally every other day for a total of seven inoculations with 3 mg/kg bodyweight of lovastatin (Cat. no. SL8750) or control DMSO. Mice were then monitored for the development of leukemia. Mice displaying signs of final stage of disease were sacrificed humanly using cervical dislocation, blood and spleens were removed, and the percentage of survival, hematocrit and spleen weight was calculated, as previously described [30].

### Statistical analysis

A statistical analysis was measured using a two tailed Student t test or a one-way ANOVA with Tukey’s post hoc test, using Prism9 GraphPad software. The P values were indicated within the figures using a standard scheme, P = < 0.05 (*), P = < 0.01 (**),P = < 0.001 (***) and P = < 0.0001 (****). Where appropriate, the data were displayed using the mean ± the SEM from at least 3 independent experiments.

## Results

### Lovastatin inhibits leukemia progression in a mouse model of erythroleukemia induced by Friend virus

**Fig. 1:**
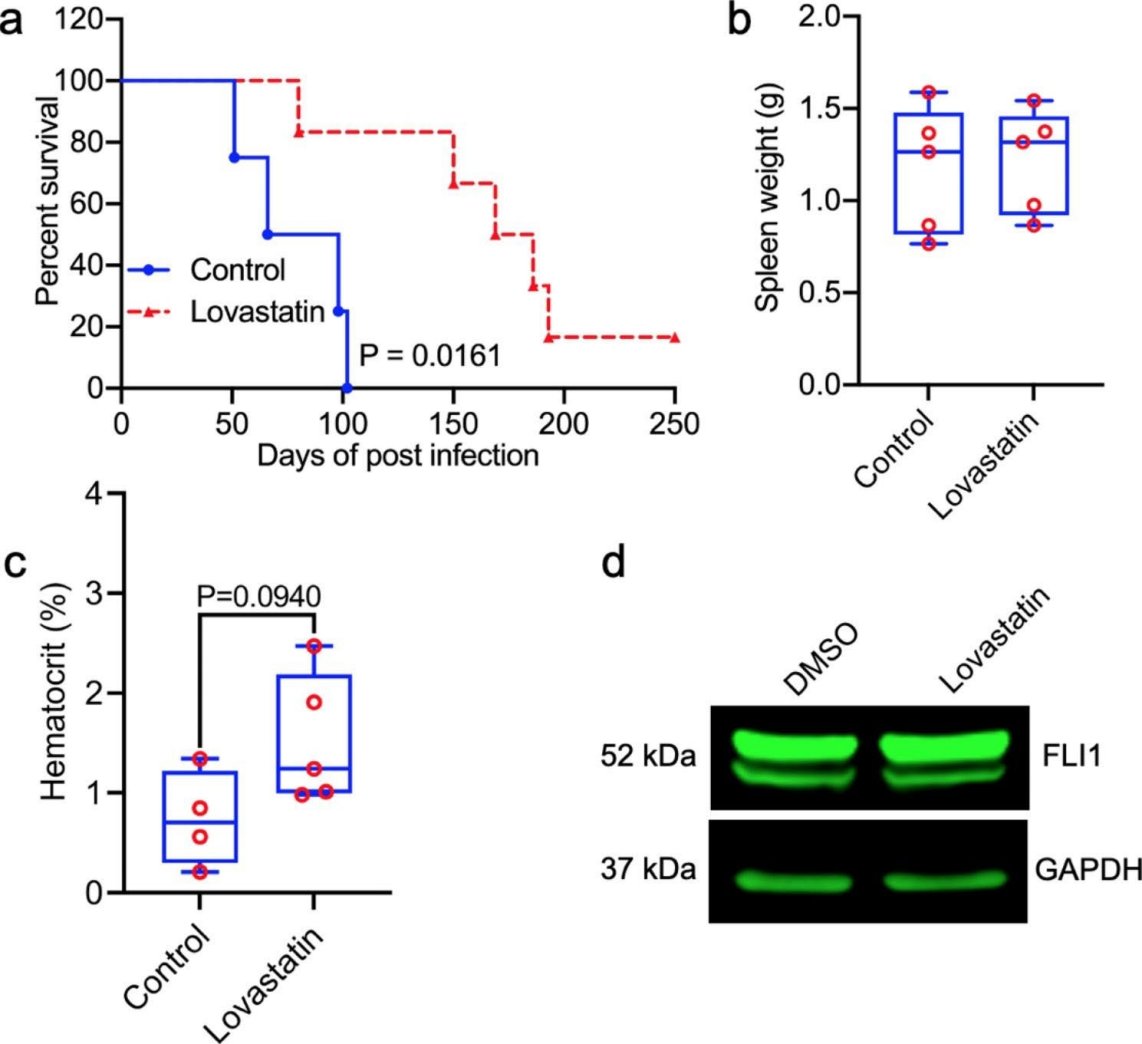
Lovastatin inhibits leukemia progression independently of FLI1. **(a)** BALB/c mice were infected with F-MuLV to induce erythroleukemia. At 5 weeks post-viral infection, mice were treated with lovastatin (3 mg/KG) every other day for 2 weeks Overall survival was used to plot a Kaplan–Meier survival curve and significance was calculated by student t-test. **(b,c)** Spleen weight **(b)** and hematocrit **(c)** of leukemic mice at the onset of morbidity. P value by two-tailed student t-test. **(d)** CB3 cells were treated with 20 µM of lovastatin for 24 h and analyzed by western blot for FLI1 expression. The full-length blots/gels are presented in **Supplementary Fig. 5**

Statins suppresses several types of cancers [32], yet the underlying mechanism remains unknown. Using a mouse model of erythroleukemia induced by Friend Murine Leukemia virus (F-MuLV), we investigated the effect of lovastatin on leukemia progression. Erythroleukemia is indeed an aggressive form of leukemia as medium survival is 3–9 month from the time of diagnosis and new drugs desperately needed for treatment of this cancer [29, 30]. Leukemias induced by F-MuLV usually appears in the spleen of infected mice around 3–5 weeks post-viral infection [30]. At 5 weeks post-viral infection, leukemic mice were treated with lovastatin (3 mg/kg), every other day for 2 weeks. Lovastatin strongly inhibited leukemia progression in these mice (Fig. 1a). At the time of death, no difference in the size of tumorigenic spleens was detected between lovastatin treated and control mice (Fig. 1b). The hematocrit values were much higher in the lovastatin treated compared to control mice, but slightly below significance (Fig. 1c**)**, suggesting a positive effect of lovastatin on red blood cell suppression during leukemia progression, which extended survival.

As Fli-1 activation is critical for murine leukemia induction by F-MuLV [33], we tested for Fli-1 levels. Lovastatin had marginal effect on Fli-1 expression (Fig. 1d) on murine erythroleukemia cell line CB3 induced by this retrovirus [29], suggesting that lovastatin inhibits leukemia progression independently of Fli-1.

### Lovastatin-mediated inhibition of leukemia cell proliferation in vitro is associated with cell cycle arrest and apoptosis

**Fig. 2:**
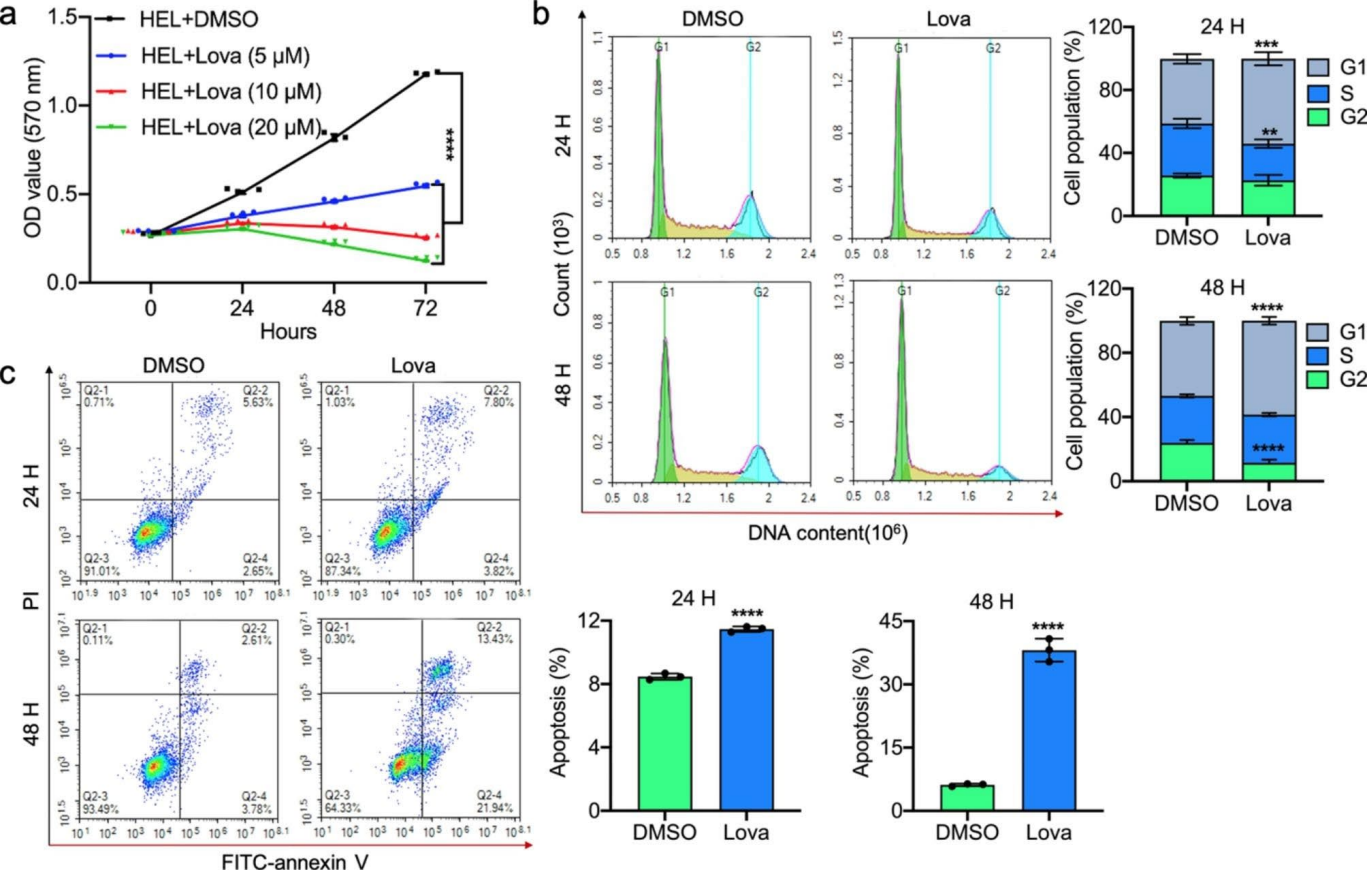
Lovastatin suppresses cell cycle and induces apoptosis in leukemic cells. **(a)** Lovastatin inhibits cell proliferation in a dose dependent manner. **(b)** HEL cells were treated with 20 µM of lovastatin for 24 and 48 h and used for cell cycle analysis. **(c)** HEL cells were treated with 20 µM of lovastatin for 24 and 48 h and subjected to apoptosis analysis

As lovastatin inhibits erythroleukemia progression in vivo (Fig. 1a), we examined the effect of this statin on erythroleukemia cell survival in culture. Lovastatin strongly inhibited the growth of human erythroleukemia cell line HEL in culture in a dose dependent manner (Fig. 2a), with an IC_50_ of 18.2 µM [18]. Similar growth inhibition is also seen in the erythroleukemia cell lines K562 and CB3, with IC_50_ of 30.2 and 35.3 µM, respectively. At 20 µM, lovastatin induced a G_1_ cell cycle arrest of HEL cells at both 24 and 48 h post-drug treatment (Fig. 2b). At this concentration, lovastatin also significantly induced apoptosis of HEL cells in culture (Fig. 2c). The above results demonstrate anti-leukemic effects of lovastatin in both tissue culture and mice.

### Lovastatin induces cholesterol biosynthesis genes in leukemic cells

**Fig. 3:**
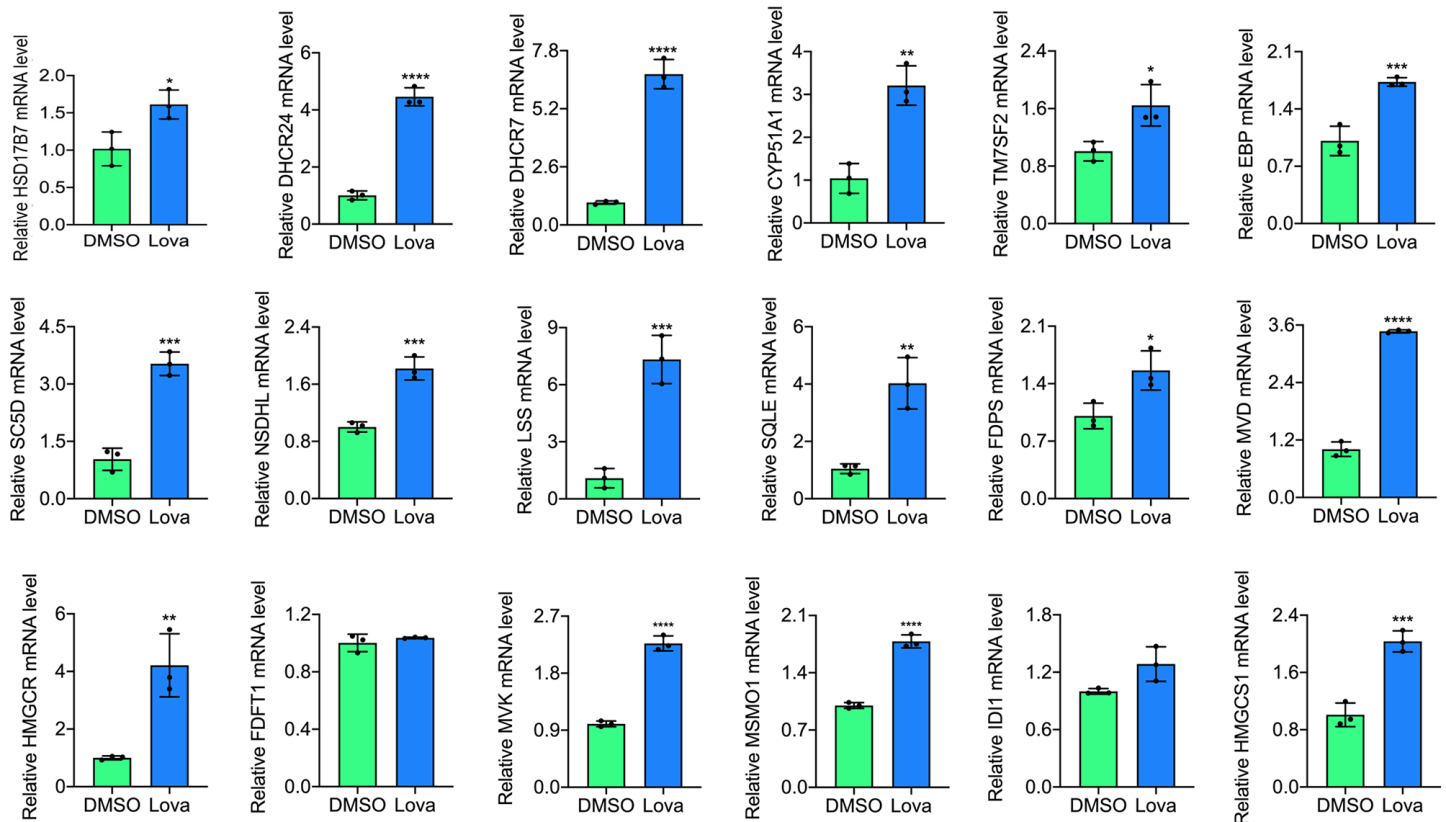
Lovastatin induces cholesterol biosynthesis gene expression in HEL cells. HEL cells were treated with 20 µM of lovastatin and used for RT-qPCR analysis for the indicated genes. P < 0.05 (*), P < 0.01 (**), P < 0.001 (***), by two-tailed student t-test

While lovastatin is known to reduce serum cholesterol by inhibiting the HMGCR (3-hydroxy-3-methylglutaryl-Coenzyme A reductase) enzyme in liver [2], the mechanism of its anti-leukemia activity is unknown. Interestingly, treatment of the human erythroleukemia cell line HEL with lovastatin (20 µM) resulted in significant transcriptional upregulation of 16 of 18 cholesterol biosynthesis genes including HMGCR (Fig. 3). Induction of HMGCR and LSS by lovastatin is also tested by western blot (**Supplementary Fig. 1a,b;** The full-length blots/gels are presented in **Supplementary Fig. 8).** Expression of FDFT1 and IDI1 was not induced by lovastatin. Interestingly, we previously demonstrated that upregulation of cholesterol biosynthesis genes by liminoid compound A1542 responsible for some of its growth inhibition activity in HEL cells line [18]. As lovastatin also inhibits cell proliferation in culture (Fig. 2a) and in vivo (Fig. 1a), higher cholesterol gene expression by this statin may be in part responsible for leukemia suppression.

Cholesterol biosynthesis gene transcription is regulated by the transcription factors SREBP1 and SREBP2 [34]. Interestingly, lovastatin significantly upregulated SREBP2 (**Supplementary Fig. 2a,b**). Lovastatin also upregulated expression of apolipoprotein A1 (APOA1), A component of high-density lipoprotein (HDL) involved in cholesterol biosynthesis (**Supplementary Fig. 2c**) [35].

**Fig. 4:**
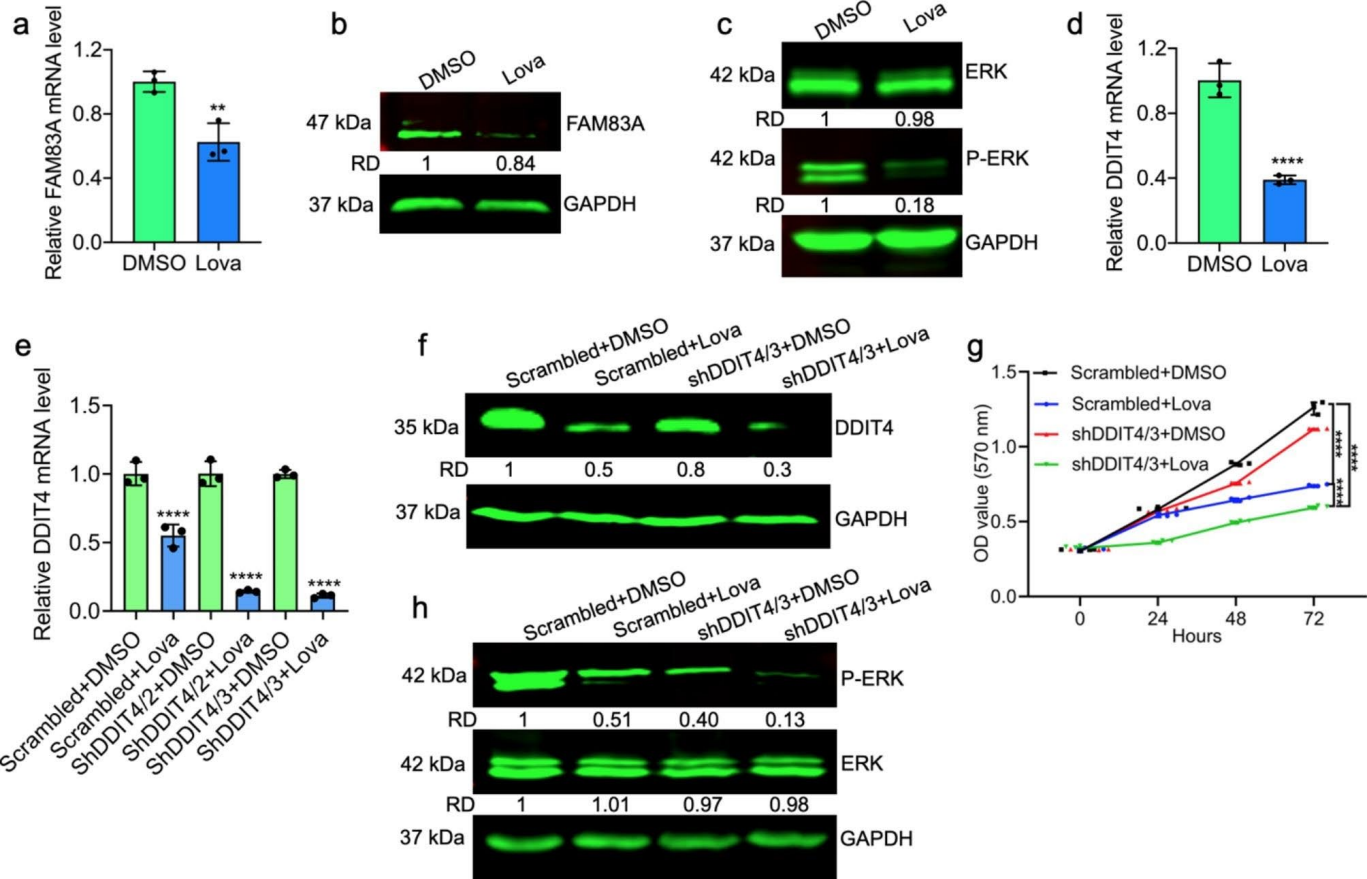
Lovastatin suppresses the expression of the FAM83A and DDIT4 oncogenes in leukemic cells. **(a,b)** HEL cells were treated with lovastatin (20 µM) and subjected to RT-qPCR and western blot analysis for expression of FAM83A. **(c)** HEL cells were treated with lovastatin for 24 h and analyzed by western blotting for expression of ERK and phospho-ERK (P-ERK). GAPDH was used as a loading control. (d) HEL cells were treated with lovastatin (20 µM) and subjected to RT-qPCR for expression of DDIT4. (e) Growth rate of shDDIT4/3 and control cells treated with or without lovastatin (20 µM) for 24 h. (f) Expression of DDIT4 and GAPDH protein in scrambled and shDDIT4/3 cells treated for 24 h with or without lovastatin (20 µM). (g) Expression of ERK, phosphor-ERK and GAPDH proteins in scrambled and shDDIT4/3 cells treated for 24 h with lovastatin (20 µM). The full-length blots/gels are presented in **Supplementary Fig. 6**

### Lovastatin suppresses expression FAM83A and DDIT4 to block the MAPK/ERK pathway

To further understand the mechanism of lovastatin growth inhibition, HEL cells were treated with lovastatin (20 µM) for 24 h and subjected to RNAseq analysis. Among genes whose expression was significantly up- or down-regulated at least 2.5 fold by lovastatin (**Supplementary Table 1**), we detected FAM83A, a known regulator of the RAS pathway, whose level was reduced by 2 folds in lovastatin-treated cells [20, 21]. Reduced expression of FAM83A by lovastatin was confirmed by RT-qPCR and western blot (Fig. 4a,b). As FAM83A regulates MAPK activity, we tested for ERK1/2 phosphorylation on Thr202/Tyr204 in lovastatin (20 µM)-treated HEL cells, and observed a strong suppression of P-ERK (Fig. 4c). Interestingly, RNAseq data also revealed strong reduction in the level of DNA damage-inducible transcript 4 (DDIT4), also known as DNA damage response 1 (REDD1). DDIT4 was shown to accelerate growth of gastric cancer cells through upregulation of MAPK [27]. Herein, we showed that lovastatin strongly suppressed DDIT4 transcription in HEL cells (Fig. 4d). To further confirm this observation, we knockdown DDIT4 using lentivirus shRNA. Two cell lines shDDIT4/2 and shDDIT4/3 were successfully generated, in which shDDIT4/3 revealed significant downregulation (Fig. 4e**)**. By western blot, while the level of DDIT4 reduced following lovastatin (20 µM) treatment, this downregulation was further increased in shDDIT4/3 cells (Fig. 4f). Cell proliferation was significantly reduced in shDDIT4/3 cells versus control, while this level further decreased when treated with lovastatin (Fig. 4g**).** Moreover, as the level of phosphor-ERK is significantly reduced in shDDIT4/3 cells, this level further decreased after treatment with lovastatin (Fig. 4h**).** These results suggest that lovastatin may suppress cell proliferation by inhibiting MAPK activity through FAM83A and DDIT4.

### Lovastatin upregulates the transcription of KLF2 that suppresses cell proliferation

**Fig. 5:**
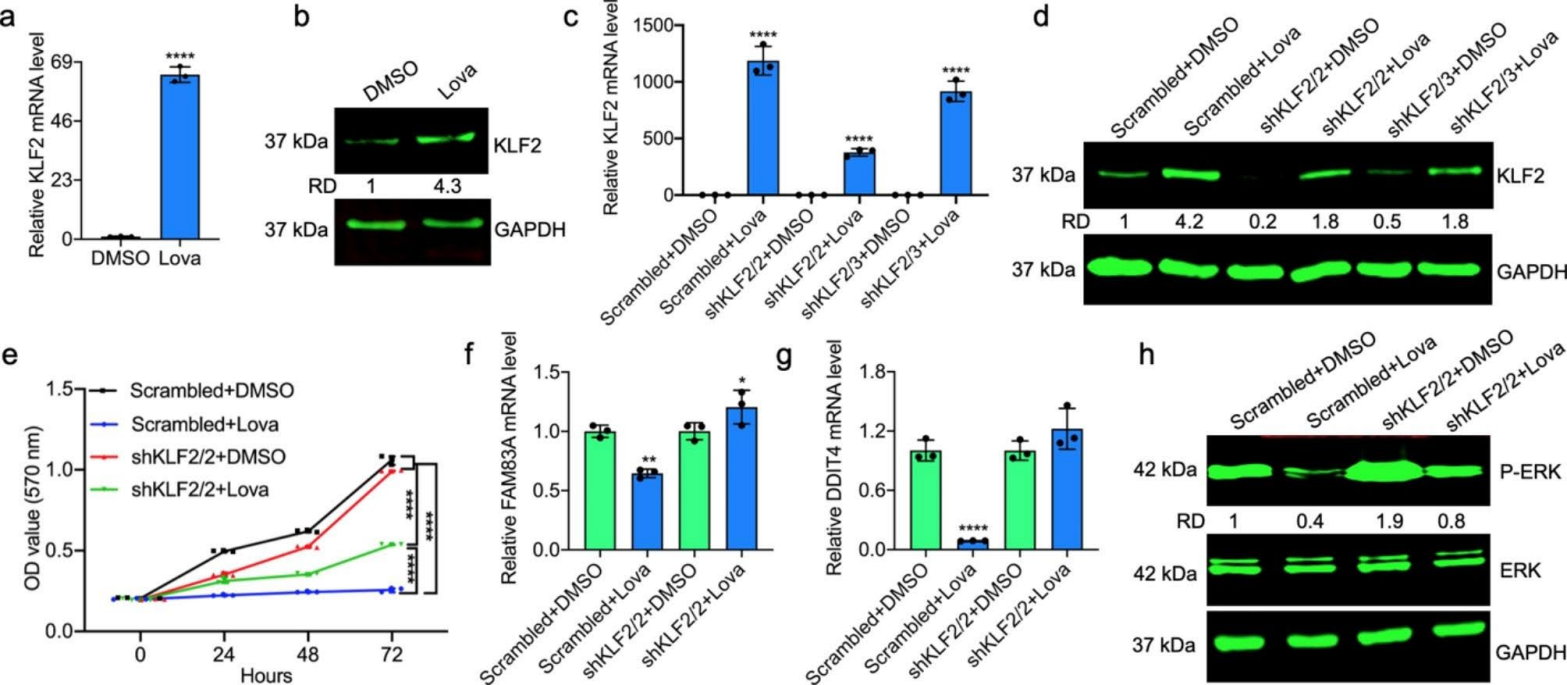
Induction of KLF2 by lovastatin suppresses MAPK/ERK through downregulation of FAM83A. **(a,b)** HEL cells were treated with 20 µM of lovastatin for 24 h and subjected to RT-qPCR **(a)** and western blot **(b)** analysis for expression of KLF2. **(c,d)** Expression of KLF2 in shKLF2/2, shKLF2/3 and scrambled control cells by RT-qPCR **(c)** and western blot **(d)**. **(e)** Growth rate of shKLF2/2 cells versus scrambled control cells for the indicated time points. The significant value between the groups is shown by brackets. **(f,g)** Expression of FAM83A **(f)** and DDIT4 **(g)** in the indicated cells after treatment for 24 h with lovastatin (20 µM). **(h)** Expression of ERK and phospho-ERK in indicated cells after treatment with lovastatin (20 µM). The full-length blots/gels are presented in **Supplementary Fig. 7**

RNAseq analysis also identified higher expression (above 400 times) of Kruppel Like Factor 2 (KLF2) in lovastatin treated HEL cells (**Supplementary Table 1**). This upregulation by lovastatin was confirmed by RT-qPCR (Fig. 5a) and Western blotting (Fig. 5b). Interestingly, KLF2 expression was significantly higher in enlarged spleens isolated from three leukemic mice treated with lovastatin versus three DMSO treated controls (**Supplementary Fig. 3;** The full-length blots/gels are presented in **Supplementary Fig. 9**). Since lovastatin suppresses cell proliferation (Fig. 2a), we examined whether this inhibition is mediated through KLF2 upregulation, as this transcription factor acts as a tumor suppressor gene [28]. Three lentivirus shRNA KLF2s (shKLF2-1, shKLF2-2 or shKLF2-3) were introduced into HEL cells. While knockdown with shKLF2-1 was not effective (data not shown), significant downregulation of KLF2 transcription was seen with shKLF2-2 and shKLF2-3 lentiviruses (Fig. 5c). As expected, KLF2 induction was significantly lower following lovastatin (20 µM) treatment in shKLF2-2 and shKLF2-3 cells by both RT-qPCR (Fig. 5c) and western blot (Fig. 5d), relative to control scrambled cells.

To detect if knockdown of KLF2 influenced cell proliferation in culture, the growth of shKLF2-2 and scrambled control cells was compared by MTT assay. Knockdown of KLF2 marginally affected the proliferation of shKLF2-2 cells compared to control cells (Fig. 5e). However, when these cells were treated with lovastatin, the growth suppression was lower in shKLF2-2 than in scrambled control cells (Fig. 5e). These results suggests that KLF2 in part is involved in growth suppression by lovastatin. Interestingly, while the level of FAM83A and DDIT4 are significantly downregulated by lovastatin in scrambled control cells, in shKLF2-2 cells this level slightly increased, although not statistically significant (Fig. 5f,g). In addition, the level of phospho-ERK is much higher in KLF2-2 than in scrambled control cells after treatment with lovastatin (Fig. 5h). These results suggest that KLF2 may suppress cell proliferation through the FAM83A/DDIT4/ERK pathway.

### KLF2 affects a subset of lovastatin induced cholesterol biosynthesis genes in leukemia cells

**Fig. 6:**
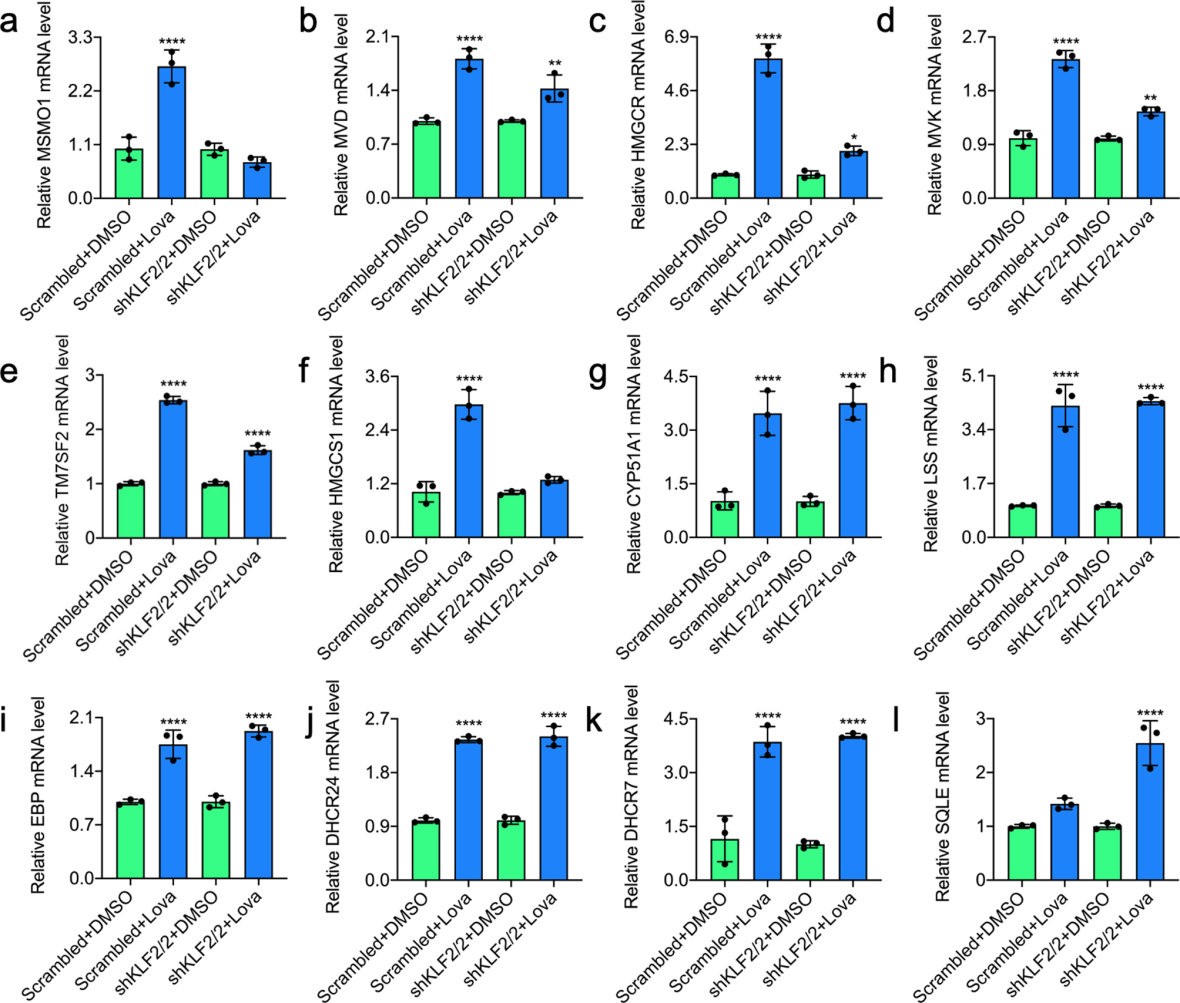
KLF2 controls the expression of a subset of cholesterol biosynthesis genes. **(a-l)** Scrambled and shKLF2/2 cells were treated for 24 h with 20 µM of lovastatin and subjected to RT-qPCR analysis for the expression of **(a) ***MSMO1*, **(b) ***MVD*, **(c) ***HMGCR*, **(d) ***MVK*, **(e) ***TM7SF2*, **(f) ***HMGCS1*, **(g) ***CYP51A1*, **(h) ***LSS*, **(i) ***EBP*, **(j) ***DHCR24*, **(k) ***DHCR7*, **(l) ***SQLE* genes.

**Fig. 7:**
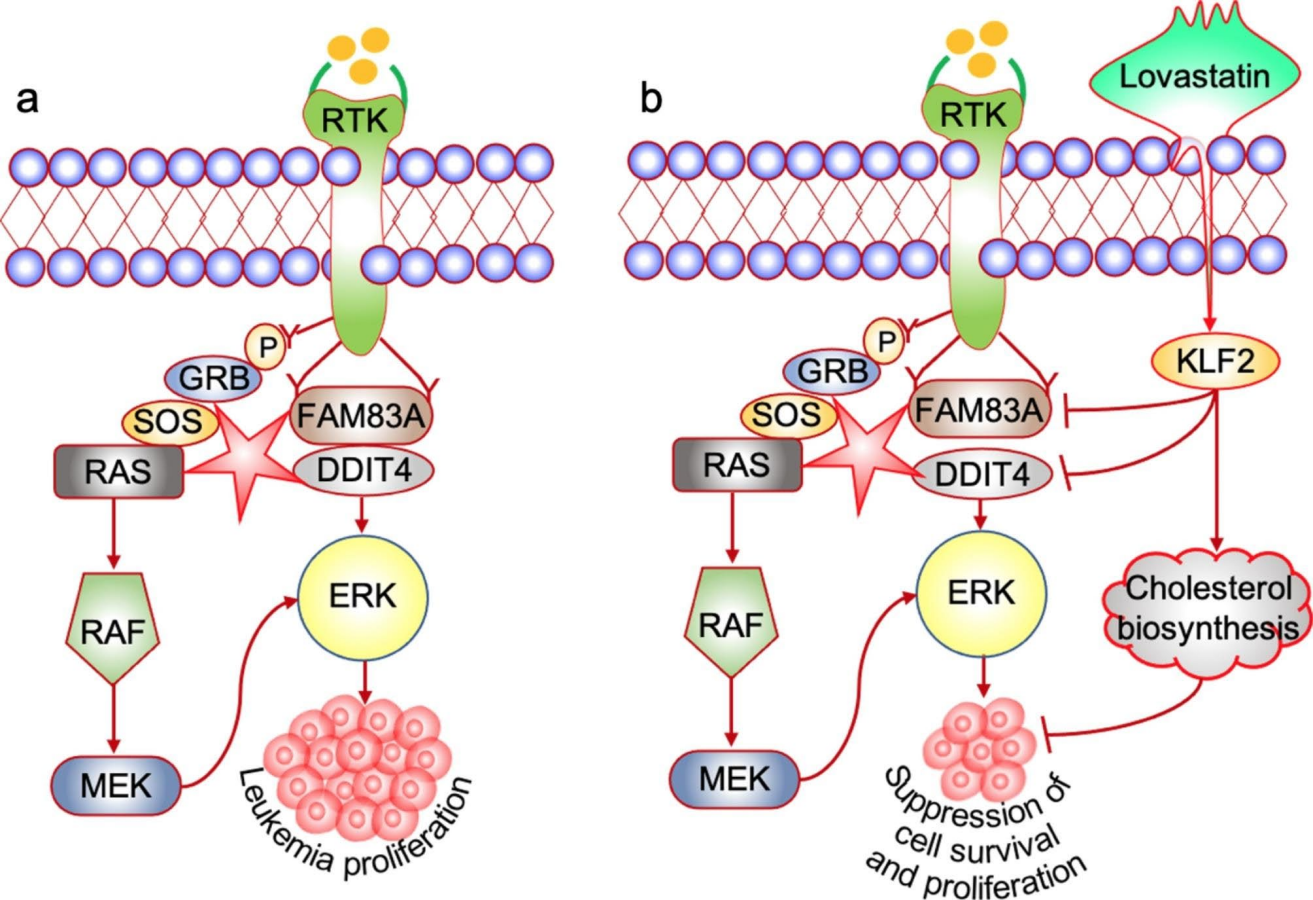
Suggested model for the effect of lovastatin on ERK1/2 via KLF2. **(a)** During leukemia proliferation, expression of FAM83A and DDIT4 promote cell proliferation by activation of ERK. **(b)** Lovastatin induces the tumor suppressor gene KLF2 leading to downregulation of FAM83A and DDIT4, and suppression of ERK. In addition, KLF2 controls the expression of certain cholesterol biosynthesis genes, which may also attenuate leukemia progression

Since cholesterol biosynthesis genes were regulated by lovastatin in leukemia cells, we examined whether this regulation is mediated through KLF2. Indeed, by examining 12 cholesterol biosynthesis genes, we showed that induction of MSMO1, MVD, HMGCR, MVK, TM7SF2 and HMGCS1 gene expression by lovastatin is much lower in shKLF2-2 cells compared to control (Fig. 6a-f). However, this induction is similar for CYP51A1, LSS, EBP, DHCR24, DHCR7 and even higher for SQLE in shKLF2-2 cells (Fig. 6g-l). In shKLF2-2 cells, while the level of SREBP1 was constant, higher expression of SREBP2 was observed, further demonstrating a partial role for KLF2 in cholesterol biosynthesis (**Supplementary Fig. 4**). In the proposed model depicted in Fig. 7a, FAM83A and DDIT4 expression promotes leukemia cell proliferation through activation of ERK. Lovastatin inhibits leukemia cell survival and proliferation in part through suppression of the FAM83A/DDIT4/ERK1/2 pathway, mediated by the induction of the tumor suppressor gene KLF2, as well as cholesterol biosynthesis genes (Fig. 7b).

## Discussion

Lovastatin, a powerful HMGCR inhibitor, is commonly used in patients to lower HDL (high-density lipoprotein). This statin compound is also associated with anti-neoplastic proliferation [19], although the mechanism is not fully understood. In this study, we show that lovastatin strongly inhibits leukemia proliferation in culture and delays erythroleukemia development in vivo. Mechanistically, Lovastatin induced the expression of cholesterol biosynthesis genes, previously shown to inhibit leukemia proliferation [18]. Moreover, lovastatin strongly induced the expression of KLF2, known to function as a tumor suppressor gene. Here we show for the first time that KLF2 exerts its inhibitory activity in part through activation of FAM83A and DDIT4, which activate the MAPK/ERK pathway [21, 27]. Overall, these results suggest that lovastatin inhibits leukemogenesis in part through KLF2 mediated FAM38A/DDIT4/ERK1/2 suppression and induction of cholesterol biosynthesis (Fig. 7).

The role of cholesterol in cancer is controversial as both high and low levels of this fat-like substance affects neoplastic progression [36]. In breast cancer, long term use of statins could promote invasive breast cancer [37]. In our previous studies, we discovered novel liminoid compounds (A1541-43) capable of inducing apoptosis in part through robust activation of cholesterol biosynthesis [18]. Here we show that lovastatin also induced expression of cholesterol biosynthesis genes in cancer cells, which may contribute as least in part to growth suppression of leukemia cells in culture and in an animal model of leukemia. As lovastatin blocks enzymatic activity of HMGCR that is critical for cholesterol biosynthesis, higher cholesterol biosynthesis may activate a compensatory mechanism by this statin. Indeed, previous studies showed that in rats with diminished basal expression of hepatic HMG-CoA reductase, animals exhibited increased sensitivity to dietary cholesterol, resulting in higher serum cholesterol [38]. In our analysis, lovastatin also induced higher levels of key cholesterol genes, Mevalonate diphosphate decarboxylast (MVD), Lanosterol synthase (LSS) and 7-dehydrocholesterol reductase (DHCR7; Fig. 3) that could compensate for cholesterol biosynthesis in the absence of HMGCR enzymatic activity. This compensatory mechanism is an interesting area of research that will require further investigation in future studies.

FAM38A is a transmembrane protein that acts as a proto-oncogene in breast cancer, downstream of the EGFR pathway [39]. FAM38A expression is critical for activation of the RAS pathway downstream of EGFR (See Fig. 7). Since lovastatin inhibits the MAPK pathway [17, 18], suppression of FAM83A, which is associated with lower ERK1/2 phosphorylation could be responsible in part for inhibition of proliferation and leukemogenicity by this statin. Moreover, the expression of another mediator of MAPK [27], DDIT4, is strongly inhibited by lovastatin. Together, these results implicate both FAM83A and DDIT4 as mediators of leukemia suppression by lovastatin.

Statins were previously reported to induce KLF2 expression, leading to activation of its downstream effectors and regulation of several pathophysiologically relevant genes [40]. Suppression of leukemia cell proliferation is indeed consistent with previously reported tumor suppressor function of KLF2, although the mechanism is still unknown [28]. Here, we showed that the induction of KLF2 by lovastatin suppressed FAM83A and DDIT4 expression, leading to lower pospho-ERK1/2 expression that likely mediates growth suppressing activity of this statin. KLF2 regulates the expression of a subset of cholesterol biosynthesis genes, that may further contribute to leukemia suppressing activity of lovastatin.

In conclusion, we show that lovastatin blocks leukemia proliferation in part through activation of cholesterol biosynthesis and induction of the transcription factor KLF2, capable of suppression the MAPKinase pathway, likely through downregulation of FAM83A and DDIT4. This study provides new insights into the mechanism of leukemia inhibition by lovastatin that could also be examined for other statins. Targeting, FAM83A and DDIT4 using small molecule drugs alone or in combination with lovastatin could be useful in treating leukemias and other cancers.

## Electronic supplementary material

Below is the link to the electronic supplementary material.

Supplementary Material 1

Supplementary Material 2

Supplementary Material 3

## Data Availability

All data generated or analysed during this study are included in this manuscript.
