# Supplementary material for: Lovastatin inhibits erythroleukemia progression through KLF2-mediated suppression of MAPK/ERK signaling
Source: BMC Cancer. 2023 Apr 4;23:306. doi: 10.1186/s12885-023-10742-4 (PMC10071686; doi:10.1186/s12885-023-10742-4)
Supplement: Supplementary file 1 — Supplementary Material 1 [file 12885_2023_10742_MOESM1_ESM.pdf]

Supplementary Table 1 | RNAsequencing data.

st_gene_id	gene_id	gene_symbol	EXP_DMSO	EXP_ Lovastatin	diffexp_log2fc_DMSO -vs-Lovastatin
G9606_28200	8362	H4C12	2.32	0	-7.857981
G9606_29511	101928589	LOC101928589	1.85	0	-7.5313815
G9606_11733	2953	GSTT2	1.82	0	-7.5077946
G9606_24237	8681	JMJD7-PLA2G4B	0.58	0	-5.857981
G9606_32443	100534592	URGCP-MRPS24	0.49	0	-5.6147098
G9606_20016	8350	H3C1	0.46	0	-5.523562
G9606_30971	440153	OR11H12	0.4	0	-5.3219281
G9606_16435	161247	FITM1	0.37	0	-5.2094534
G9606_23903	389125	MUSTN1	0.37	0	-5.2094534
G9606_35077	135458	HUS1B	0.36	0	-5.169925
G9606_8263	2543	GAGE1	0.34	0	-5.0874628
G9606_12661	102723360	LOC102723360	0.34	0	-5.0874628
G9606_17829	6346	CCL1	0.33	0	-5.0443941
G9606_4005	102724334	LOC102724334	0.33	0	-5.0443941
G9606_32654	645051	GAGE13	0.29	0	-4.857981
G9606_68939	114118903	ARHGAP11A-SCG5	0.27	0	-4.7548875
G9606_23549	6462	SHBG	0.27	0	-4.7548875
G9606_12503	140711	TLDC2	0.27	0	-4.7548875
G9606_34219	93659	CGB5	0.26	0	-4.7004397
G9606_20615	51327	AHSP	0.25	0	-4.6438562
G9606_27920	56158	TEX12	0.25	0	-4.6438562
G9606_26299	7123	CLEC3B	0.24	0	-4.5849625
G9606_21109	283869	NPW	0.22	0	-4.4594316
G9606_13328	102724652	CRYAA2	0.21	0	-4.3923174
G9606_8904	3972	LHB	0.21	0	-4.3923174
G9606_8340	203569	PAGE2	0.2	0	-4.3219281
G9606_26978	51480	VCX2	0.2	0	-4.3219281
G9606_12317	100526760	ABHD14A-ACY1	0.19	0	-4.2479275
G9606_7227	2253	FGF8	0.18	0	-4.169925
G9606_6061	114928	GPRASP2	0.18	0	-4.169925
G9606_20101	6236	RRAD	0.18	0	-4.169925
G9606_20922	29802	VPREB3	0.18	0	-4.169925
G9606_10605	353500	BMP8A	0.17	0	-4.0874628
G9606_3840	1159	CKMT1B	0.17	0	-4.0874628
G9606_32570	2980	GUCA2A	0.17	0	-4.0874628
G9606_35658	474382	H2AB1	0.17	0	-4.0874628
G9606_24313	253582	TMEM244	0.17	0	-4.0874628
G9606_33017	100170765	ERICH4	0.16	0	-4

G9606_27496	7379	UPK2	0.16	0	-4
G9606_7290	4935	GPR143	0.15	0	-3.9068906
G9606_22896	728361	OVOL3	0.15	0	-3.9068906
G9606_393	126969	SLC44A3	0.15	0	-3.9068906
G9606_35610	729201	SPACA5B	0.15	0	-3.9068906
G9606_17448	100653133	LOC100653133	0.14	0	-3.8073549
G9606_10282	149499	LRRC71	0.14	0	-3.8073549
G9606_9169	27334	P2RY10	0.14	0	-3.8073549
G9606_12124	763	CA5A	0.13	0	-3.7004397
G9606_26138	115811	IQCD	0.13	0	-3.7004397
G9606_31000	6703	SPRR2D	0.13	0	-3.7004397
G9606_14327	7125	TNNC2	0.13	0	-3.7004397
G9606_3040	7253	TSHR	0.13	0	-3.7004397
G9606_6120	11326	VSIG4	0.13	0	-3.7004397
G9606_20572	100874261	CCDC200	0.12	0	-3.5849625
G9606_1934	8900	CCNA1	0.12	0	-3.5849625
G9606_11140	100529144	CORO7-PAM16	0.12	0	-3.5849625
G9606_14223	54097	FAM3B	0.12	0	-3.5849625
G9606_17299	3126	HLA-DRB4	0.12	0	-3.5849625
G9606_28903	7044	LEFTY2	0.12	0	-3.5849625
G9606_2196	149998	LIPI	0.12	0	-3.5849625
G9606_31805	10744	PTTG2	0.12	0	-3.5849625
G9606_10102	137902	PXDNL	0.12	0	-3.5849625
G9606_25558	6036	RNASE2	0.12	0	-3.5849625
G9606_5751	342933	ZSCAN5B	0.12	0	-3.5849625
G9606_33007	30848	CTAG2	0.11	0	-3.4594316
G9606_34859	349334	FOXD4L4	0.11	0	-3.4594316
G9606_5665	9630	GNA14	0.11	0	-3.4594316
G9606_10104	171558	PTCRA	0.11	0	-3.4594316
G9606_4438	727940	RHOXF2B	0.11	0	-3.4594316
G9606_20563	6101	RP1	0.11	0.01	-3.4594316
G9606_30263	219539	YPEL4	0.11	0	-3.4594316
G9606_31430	27299	ADAMDEC1	0.1	0	-3.3219281
G9606_35892	267020	ATP5MGL	0.1	0	-3.3219281
G9606_30720	525	ATP6V1B1	0.1	0	-3.3219281
G9606_3936	80125	CCDC33	0.1	0	-3.3219281
G9606_26256	1366	CLDN7	0.1	0	-3.3219281
G9606_28087	1437	CSF2	0.1	0	-3.3219281
G9606_26104	8214	DGCR6	0.1	0	-3.3219281
G9606_539	84332	DYDC2	0.1	0	-3.3219281
G9606_13815	730394	GTF2H2C_2	0.1	0	-3.3219281
G9606_28514	100506164	HSFX1	0.1	0	-3.3219281
G9606_28289	254050	LRRC43	0.1	0	-3.3219281

G9606_10244	135250	RAET1E	0.1	0	-3.3219281
G9606_295	11262	SP140	0.1	0	-3.3219281
G9606_6631	130733	TMEM178A	0.1	0	-3.3219281
G9606_29820	339453	TMEM240	0.1	0	-3.3219281
G9606_11174	7476	WNT7A	0.29	0.03	-3.2730185
G9606_34182	147670	SMIM17	0.19	0.02	-3.2479275
G9606_526	72	ACTG2	0.09	0	-3.169925
G9606_22040	100130520	CD300H	0.09	0	-3.169925
G9606_26180	387885	CFAP73	0.09	0	-3.169925
G9606_5383	10170	DHRS9	0.09	0	-3.169925
G9606_14928	2028	ENPEP	0.09	0	-3.169925
G9606_12710	644145	EXOC1L	0.09	0	-3.169925
G9606_9210	2352	FOLR3	0.09	0	-3.169925
G9606_34793	26330	GAPDHS	0.09	0	-3.169925
G9606_24952	254910	LCE5A	0.09	0	-3.169925
G9606_11041	105370705	LOC105370705	0.09	0	-3.169925
G9606_30143	4109	MAGEA10	0.09	0	-3.169925
G9606_4521	4311	MME	0.09	0.01	-3.169925
G9606_12817	128506	OCSTAMP	0.09	0	-3.169925
G9606_9471	56127	PCDHB9	0.09	0.01	-3.169925
G9606_7755	254122	SNX32	0.09	0	-3.169925
G9606_29188	608	TNFRSF17	0.09	0	-3.169925
G9606_7003	7293	TNFRSF4	0.09	0	-3.169925
G9606_3757	51733	UPB1	0.27	0.03	-3.169925
G9606_23502	51351	ZNF117	0.09	0	-3.169925
G9606_29991	56265	CPXM1	0.25	0.03	-3.0588937
G9606_23371	644974	ALG1L2	0.08	0	-3
G9606_25804	145407	ARMH4	0.08	0.01	-3
G9606_34566	147685	C19orf18	0.08	0	-3
G9606_31387	124599	CD300LB	0.08	0	-3
G9606_6564	146894	CD300LG	0.08	0	-3
G9606_18145	1410	CRYAB	0.08	0	-3
G9606_26393	100507170	CT47A12	0.08	0.01	-3
G9606_22642	728082	CT47A3	0.08	0.01	-3
G9606_10615	728072	CT47A5	0.08	0.01	-3
G9606_28886	728042	CT47A9	0.08	0.01	-3
G9606_9746	60401	EDA2R	0.16	0.02	-3
G9606_10031	84553	FAXC	0.08	0	-3
G9606_14158	2651	GCNT2	0.08	0	-3
G9606_3258	389396	GLYATL3	0.08	0	-3
G9606_33819	7923	HSD17B8	0.08	0	-3
G9606_5736	10964	IFI44L	0.08	0	-3
G9606_18193	101929692	LOC101929692	0.08	0	-3

G9606_17004	643802	LOC643802	0.08	0	-3
G9606_32114	4113	MAGEB2	0.08	0	-3
G9606_17569	25834	MGAT4C	0.08	0	-3
G9606_26233	282770	OR10AG1	0.08	0	-3
G9606_20387	26693	OR2V1	0.08	0	-3
G9606_33032	170679	PSORS1C1	0.08	0	-3
G9606_11272	374897	SBSN	0.08	0	-3
G9606_1534	348378	SHISAL2A	0.08	0	-3
G9606_8619	57413	TMIGD3	0.08	0	-3
G9606_22202	26609	VCX	0.08	0	-3
G9606_6496	440822	PIWIL3	0.23	0.03	-2.9385995
G9606_29328	100130311	C17orf107	0.07	0	-2.8073549
G9606_9659	942	CD86	0.07	0	-2.8073549
G9606_25936	140947	DCANP1	0.07	0	-2.8073549
G9606_35185	347516	DGAT2L6	0.07	0	-2.8073549
G9606_6576	150350	ENTHD1	0.07	0	-2.8073549
G9606_17836	131890	GRK7	0.07	0.01	-2.8073549
G9606_17535	3274	HRH2	0.07	0.01	-2.8073549
G9606_12901	146433	IL34	0.07	0	-2.8073549
G9606_16962	284359	IZUMO1	0.07	0	-2.8073549
G9606_34579	113730	KLHDC7B	0.07	0	-2.8073549
G9606_3001	120071	LARGE2	0.07	0	-2.8073549
G9606_452	105376525	LOC105376525	0.07	0	-2.8073549
G9606_33980	105376722	LOC105376722	0.07	0	-2.8073549
G9606_4618	646627	LYPD8	0.07	0	-2.8073549
G9606_25229	4880	NPPC	0.07	0	-2.8073549
G9606_29881	122748	OR11H6	0.07	0	-2.8073549
G9606_14830	653781	POTEJ	0.07	0	-2.8073549
G9606_4652	5590	PRKCZ	0.07	0	-2.8073549
G9606_31568	5697	PYY	0.07	0	-2.8073549
G9606_9204	57402	S100A14	0.07	0	-2.8073549
G9606_24717	85477	SCIN	0.07	0.01	-2.8073549
G9606_7045	6444	SGCD	0.07	0	-2.8073549
G9606_15695	57586	SYT13	0.07	0.01	-2.8073549
G9606_21597	259294	TAS2R19	0.07	0	-2.8073549
G9606_32338	85012	TCEAL3	0.07	0	-2.8073549
G9606_28559	7045	TGFBI	0.07	0	-2.8073549
G9606_23227	391123	VSIG8	0.07	0	-2.8073549
G9606_6776	126248	WDR88	0.07	0	-2.8073549
G9606_14392	30832	ZNF354C	0.07	0.01	-2.8073549
G9606_15076	107986762	LOC107986762	0.19	0.03	-2.662965
G9606_9725	8785	MATN4	0.19	0.03	-2.662965
G9606_11763	88	ACTN2	0.06	0.01	-2.5849625

G9606_30962	383	ARG1	0.06	0	-2.5849625
G9606_5775	50649	ARHGEF4	0.12	0.02	-2.5849625
G9606_34	116969	ART5	0.06	0	-2.5849625
G9606_21374	79369	B3GNT4	0.06	0	-2.5849625
G9606_11311	646457	C19orf67	0.06	0	-2.5849625
G9606_3380	90557	CCDC74A	0.06	0	-2.5849625
G9606_15350	1236	CCR7	0.06	0	-2.5849625
G9606_21170	1443	CSH2	0.06	0	-2.5849625
G9606_8392	66002	CYP4F12	0.06	0	-2.5849625
G9606_14113	400830	DEFB132	0.06	0	-2.5849625
G9606_21250	286380	FOXD4L3	0.06	0	-2.5849625
G9606_16241	2525	FUT3	0.06	0	-2.5849625
G9606_26361	439996	IFIT1B	0.06	0	-2.5849625
G9606_9253	11185	INMT	0.12	0.02	-2.5849625
G9606_5187	3742	KCNA6	0.06	0	-2.5849625
G9606_2308	23415	KCNH4	0.06	0	-2.5849625
G9606_21013	105371932	LOC105371932	0.06	0	-2.5849625
G9606_5041	107986910	LOC107986910	0.06	0	-2.5849625
G9606_11388	158809	MAGEB6	0.06	0	-2.5849625
G9606_24160	100129480	MKRN2OS	0.06	0	-2.5849625
G9606_17077	253827	MSRB3	0.06	0	-2.5849625
G9606_1918	284958	NT5DC4	0.06	0	-2.5849625
G9606_15712	4909	NTF4	0.06	0	-2.5849625
G9606_13243	55200	PLEKHG6	0.12	0.02	-2.5849625
G9606_12745	343035	RD3	0.06	0	-2.5849625
G9606_8896	346673	STRA8	0.06	0	-2.5849625
G9606_8478	56849	TCEAL7	0.06	0	-2.5849625
G9606_28247	25823	TPSG1	0.06	0	-2.5849625
G9606_411	341346	SMCO2	0.41	0.07	-2.5501971
G9606_3693	3292	HSD17B1	0.64	0.11	-2.5405684
G9606_24358	93145	OLFM2	0.23	0.04	-2.523562
G9606_8050	631	BFSP1	0.03	0.17	2.50250034
G9606_12225	2793	GNGT2	0.08	0.46	2.52356196
G9606_32324	143689	PIWIL4	0.04	0.23	2.52356196
G9606_4201	84448	ABLIM2	0	0.06	2.5849625
G9606_26532	474	ATOH1	0	0.06	2.5849625
G9606_24365	50617	ATP6V0A4	0	0.06	2.5849625
G9606_33232	655	BMP7	0	0.06	2.5849625
G9606_19636	388759	C1orf229	0	0.06	2.5849625
G9606_7797	92291	CAPN13	0	0.06	2.5849625
G9606_25089	147372	CCBE1	0.01	0.06	2.5849625
G9606_1348	79846	CFAP69	0	0.06	2.5849625
G9606_11087	387837	CLEC12B	0	0.06	2.5849625

G9606_23742	1409	CRYAA	0	0.06	2.5849625
G9606_21950	90288	EFCAB12	0.01	0.06	2.5849625
G9606_917	377841	ENTPD8	0	0.06	2.5849625
G9606_2122	388799	FAM209B	0	0.06	2.5849625
G9606_17532	100288801	FRG2C	0	0.06	2.5849625
G9606_13429	3250	HPR	0	0.06	2.5849625
G9606_33553	56832	IFNK	0	0.06	2.5849625
G9606_23731	3589	IL11	0	0.06	2.5849625
G9606_15201	27177	IL36B	0	0.06	2.5849625
G9606_9289	126306	JSRP1	0	0.06	2.5849625
G9606_21555	3766	KCNJ10	0.01	0.06	2.5849625
G9606_3578	346689	KLRG2	0	0.06	2.5849625
G9606_9472	54900	LAX1	0	0.06	2.5849625
G9606_981	107984153	LOC107984153	0.01	0.06	2.5849625
G9606_1753	22808	MRAS	0	0.06	2.5849625
G9606_28216	579	NKX3-2	0	0.06	2.5849625
G9606_9886	56923	NMUR2	0	0.06	2.5849625
G9606_4147	389643	NUGGC	0	0.06	2.5849625
G9606_28512	158046	NXNL2	0	0.06	2.5849625
G9606_14379	8612	PLPP2	0	0.06	2.5849625
G9606_22112	388199	PRR25	0	0.06	2.5849625
G9606_16260	83998	REG4	0	0.06	2.5849625
G9606_17699	5999	RGS4	0	0.06	2.5849625
G9606_18831	90203	SNX21	0	0.06	2.5849625
G9606_31325	6781	STC1	0.01	0.06	2.5849625
G9606_24284	221711	SYCP2L	0	0.06	2.5849625
G9606_12456	23546	SYNGR4	0.08	0.48	2.5849625
G9606_20453	100529257	SYNJ2BP-COX16	0	0.06	2.5849625
G9606_25502	7069	THRSP	0	0.06	2.5849625
G9606_16593	27283	TINAG	0	0.06	2.5849625
G9606_2061	494513	PJVK	0.1	0.62	2.63226822
G9606_29163	9080	CLDN9	0.04	0.25	2.64385619
G9606_3949	346653	FAM71F2	0.03	0.19	2.66296501
G9606_28180	107987233	LOC107987233	0.07	0.45	2.68449817
G9606_35494	440503	PLIN5	0.02	0.13	2.70043972
G9606_26400	59335	PRDM12	0.02	0.13	2.70043972
G9606_27866	128434	VSTM2L	0.03	0.2	2.73696559
G9606_27293	101060389	TBC1D3D	0.04	0.27	2.7548875
G9606_29857	137872	ADHFE1	0	0.07	2.80735492
G9606_4678	57085	AGTRAP	0	0.07	2.80735492
G9606_12450	8419	BFSP2	0	0.07	2.80735492
G9606_34987	110806296	C1orf232	0	0.07	2.80735492
G9606_26121	79935	CCNP	0	0.07	2.80735492

G9606_1971	1438	CSF2RA	0	0.07	2.80735492
G9606_21841	145781	GCOM1	0	0.07	2.80735492
G9606_16990	3231	HOXD1	0	0.07	2.80735492
G9606_24974	3960	LGALS4	0	0.07	2.80735492
G9606_29324	107985734	LOC107985734	0	0.07	2.80735492
G9606_17760	112267951	LOC112267951	0	0.07	2.80735492
G9606_8753	225689	MAPK15	0	0.07	2.80735492
G9606_1083	346606	MOGAT3	0	0.07	2.80735492
G9606_25934	4607	MYBPC3	0	0.07	2.80735492
G9606_32717	392517	NCBP2L	0	0.07	2.80735492
G9606_12343	4939	OAS2	0	0.07	2.80735492
G9606_35544	119774	OR52K2	0	0.07	2.80735492
G9606_6028	5734	PTGER4	0	0.07	2.80735492
G9606_21086	282808	RAB40AL	0	0.07	2.80735492
G9606_8701	388015	RTL1	0.01	0.07	2.80735492
G9606_32350	100132963	SMIM9	0	0.07	2.80735492
G9606_19000	165904	XIRP1	0	0.07	2.80735492
G9606_10793	100129842	ZNF737	0	0.07	2.80735492
G9606_2737	162962	ZNF836	0.01	0.07	2.80735492
G9606_29174	81569	ACTL8	0.04	0.29	2.857981
G9606_6963	56917	MEIS3	0.04	0.29	2.857981
G9606_29675	353497	POLN	0.05	0.39	2.96347412
G9606_20778	122481	AK7	0.04	0.32	3
G9606_7470	146562	C16orf71	0	0.08	3
G9606_22996	919	CD247	0	0.08	3
G9606_6680	497190	CLEC18B	0	0.08	3
G9606_30449	125965	COX6B2	0	0.08	3
G9606_26383	339768	ESPNL	0.01	0.08	3
G9606_30227	84734	FAM167B	0	0.08	3
G9606_6895	92086	GGTLC1	0	0.08	3
G9606_14774	2844	GPR21	0.01	0.08	3
G9606_20007	285643	KIF4B	0.01	0.08	3
G9606_22592	105376678	LOC105376678	0	0.08	3
G9606_16445	29881	NPC1L1	0.01	0.08	3
G9606_23195	152816	ODAPH	0	0.08	3
G9606_14074	127608	OR2AJ1	0	0.08	3
G9606_35176	391192	OR2L3	0	0.08	3
G9606_14246	79317	OR4K5	0	0.08	3
G9606_19324	64714	PDIA2	0	0.08	3
G9606_30903	5453	POU3F1	0	0.08	3
G9606_30199	145226	RDH12	0.03	0.24	3
G9606_9344	7782	SLC30A4	0.01	0.08	3
G9606_27134	399668	SMIM10L2A	0.01	0.08	3

G9606_8565	9901	SRGAP3	0	0.08	3
G9606_18156	259292	TAS2R46	0	0.08	3
G9606_25037	646799	ZAR1L	0	0.08	3
G9606_33168	7712	ZNF157	0.02	0.17	3.08746284
G9606_2788	1316	KLF6	0.79	7	3.14743036
G9606_35936	566	AZU1	0	0.09	3.169925
G9606_33560	100131378	C11orf91	0	0.09	3.169925
G9606_30401	1562	CYP2C18	0	0.09	3.169925
G9606_30882	2634	GBP2	0.01	0.09	3.169925
G9606_1954	3002	GZMB	0	0.09	3.169925
G9606_27798	474381	H2AB2	0	0.09	3.169925
G9606_31841	83740	H2AB3	0	0.09	3.169925
G9606_20180	3805	KIR2DL4	0	0.09	3.169925
G9606_5531	136259	KLF14	0	0.09	3.169925
G9606_22278	200634	KRTCAP3	0	0.09	3.169925
G9606_8277	101929400	LOC101929400	0	0.09	3.169925
G9606_26272	102724965	LOC102724965	0	0.09	3.169925
G9606_21413	107984043	LOC107984043	0	0.09	3.169925
G9606_4995	130752	MDH1B	0	0.09	3.169925
G9606_16004	5346	PLIN1	0	0.09	3.169925
G9606_18983	154197	PNLDC1	0	0.09	3.169925
G9606_15384	5626	PROP1	0	0.09	3.169925
G9606_4720	729974	RFPL4AL1	0	0.09	3.169925
G9606_6092	51151	SLC45A2	0	0.09	3.169925
G9606_32461	285051	STPG4	0	0.09	3.169925
G9606_11287	199964	TMEM61	0	0.09	3.169925
G9606_21888	399939	TRIM49D1	0	0.09	3.169925
G9606_21639	164153	UBL4B	0	0.09	3.169925
G9606_31716	978	CDA	0	0.1	3.32192809
G9606_29108	1357	CPA1	0	0.1	3.32192809
G9606_35344	1815	DRD4	0	0.1	3.32192809
G9606_14554	51233	DRICH1	0	0.1	3.32192809
G9606_12475	3266	ERAS	0	0.1	3.32192809
G9606_30884	653404	FOXD4L6	0	0.1	3.32192809
G9606_9660	100130086	HSFX2	0	0.1	3.32192809
G9606_3296	113220	KIF12	0	0.1	3.32192809
G9606_29126	4878	NPPA	0	0.1	3.32192809
G9606_26152	138429	PIP5KL1	0.02	0.2	3.32192809
G9606_19142	124912	SPACA3	0	0.1	3.32192809
G9606_565	201305	SPNS3	0	0.1	3.32192809
G9606_14795	727837	SSX2B	0	0.1	3.32192809
G9606_8195	120379	PIH1D2	0.07	0.72	3.36257008
G9606_32879	1215	CMA1	0	0.11	3.45943162

G9606_27849	8506	CNTNAP1	0.01	0.11	3.45943162
G9606_182	79852	EPHX3	0	0.11	3.45943162
G9606_30586	26290	GALNT8	0	0.11	3.45943162
G9606_7129	3821	KLRC1	0	0.11	3.45943162
G9606_16199	102724474	LOC102724474	0	0.11	3.45943162
G9606_34862	10894	LYVE1	0.02	0.22	3.45943162
G9606_21586	57134	MAN1C1	0	0.11	3.45943162
G9606_35072	116729	PPP1R27	0	0.11	3.45943162
G9606_24047	2837	UTS2R	0	0.11	3.45943162
G9606_28700	6347	CCL2	0	0.12	3.5849625
G9606_3320	55679	LIMS2	0	0.12	3.5849625
G9606_27092	145873	MESP2	0	0.12	3.5849625
G9606_14444	4256	MGP	0	0.12	3.5849625
G9606_9894	6588	SLN	0	0.12	3.5849625
G9606_1485	83890	SPATA9	0	0.12	3.5849625
G9606_11604	729767	CEACAM18	0	0.13	3.70043972
G9606_21843	1081	CGA	0	0.13	3.70043972
G9606_17040	10669	CGREF1	0	0.13	3.70043972
G9606_16723	128821	CST9L	0	0.13	3.70043972
G9606_32270	2695	GIP	0	0.13	3.70043972
G9606_11232	5947	RBP1	0	0.13	3.70043972
G9606_11354	440184	CCDC196	0	0.14	3.80735492
G9606_17479	157807	CLVS1	0	0.14	3.80735492
G9606_20548	493861	EID3	0	0.14	3.80735492
G9606_639	728441	GGT2	0	0.14	3.80735492
G9606_35592	245802	MS4A6E	0	0.14	3.80735492
G9606_29451	64648	SPANXD	0	0.14	3.80735492
G9606_32720	55350	VNN3	0	0.14	3.80735492
G9606_17147	343	AQP8	0	0.15	3.9068906
G9606_22612	929	CD14	0	0.15	3.9068906
G9606_14346	641700	ECSCR	0	0.15	3.9068906
G9606_9625	10361	NPM2	0	0.15	3.9068906
G9606_36037	5222	PGA5	0	0.15	3.9068906
G9606_22809	643596	RNF224	0	0.15	3.9068906
G9606_26045	143941	TTC36	0	0.15	3.9068906
G9606_3106	165545	DQX1	0.03	0.47	3.96962635
G9606_34594	56311	ANKRD7	0	0.16	4
G9606_24513	3112	HLA-DOB	0	0.16	4
G9606_14434	81470	OR2G2	0	0.16	4
G9606_35994	2810	SFN	0	0.16	4
G9606_31241	81469	OR2G3	0	0.17	4.08746284
G9606_7877	102724127	TP53TG3F	0	0.17	4.08746284
G9606_10018	255119	CFAP299	0	0.18	4.169925

G9606_28791	168537	GIMAP7	0	0.18	4.169925
G9606_10733	2939	GSTA2	0	0.18	4.169925
G9606_29808	100310846	ANKRD61	0	0.19	4.24792751
G9606_12603	126393	HSPB6	0	0.19	4.24792751
G9606_15614	254773	LYG2	0	0.19	4.24792751
G9606_19558	100532736	MICOS10-NBL1	0	0.19	4.24792751
G9606_19102	29895	MYLPF	0	0.19	4.24792751
G9606_12896	27232	GNMT	0	0.2	4.32192809
G9606_11115	402381	SOHLH1	0	0.2	4.32192809
G9606_20503	441168	CALHM6	0	0.21	4.39231742
G9606_15388	121355	GTSF1	0	0.21	4.39231742
G9606_13872	145376	PPP1R36	0	0.21	4.39231742
G9606_18943	54210	TREM1	0	0.21	4.39231742
G9606_12808	728734	NPIPB8	0.05	1.06	4.40599236
G9606_8236	57818	G6PC2	0	0.22	4.45943162
G9606_16420	100528032	KLRC4-KLRK1	0	0.22	4.45943162
G9606_9177	105378949	LOC105378949	0	0.22	4.45943162
G9606_26189	100652777	LOC100652777	0	0.23	4.52356196
G9606_3822	27122	DKK3	0	0.24	4.5849625
G9606_33982	149643	SPATA45	0	0.24	4.5849625
G9606_33420	26212	OR2B6	0	0.25	4.64385619
G9606_11391	283927	NUDT7	0	0.27	4.7548875
G9606_20194	283106	CSNK2A3	0	0.29	4.857981
G9606_18624	644186	SYCE3	0	0.3	4.9068906
G9606_34271	1469	CST1	0	0.34	5.08746284
G9606_16554	51027	BOLA1	0	0.36	5.169925
G9606_2548	6447	SCG5	0	0.38	5.24792751
G9606_20018	342977	NANOS3	0	0.4	5.32192809
G9606_19726	335	APOA1	0	0.44	5.45943162
G9606_19962	5149	PDE6H	0	0.48	5.5849625
G9606_13338	101927345	LOC101927345	0	0.5	5.64385619
G9606_31661	5730	PTGDS	0	0.52	5.70043972
G9606_27855	106865373	GET1-SH3BGR	0	0.57	5.83289001
G9606_35106	729428	GAGE12B	0	0.58	5.857981
G9606_21500	102724219	LOC102724219	0	0.59	5.88264305
G9606_17216	100527949	GIMAP1-GIMAP5	0	0.63	5.97727992
G9606_32396	10365	KLF2	0.13	9.02	6.11654391
G9606_29192	8368	H4C13	0	0.94	6.55458885
G9606_24200	100137049	PLA2G4B	0	1.48	7.20945337
