# Supplementary material for: Lovastatin inhibits erythroleukemia progression through KLF2-mediated suppression of MAPK/ERK signaling
Source: BMC Cancer. 2023 Apr 4;23:306. doi: 10.1186/s12885-023-10742-4 (PMC10071686; doi:10.1186/s12885-023-10742-4)
Supplement: Supplementary file 2 — Supplementary Material 2 [file 12885_2023_10742_MOESM2_ESM.pdf]

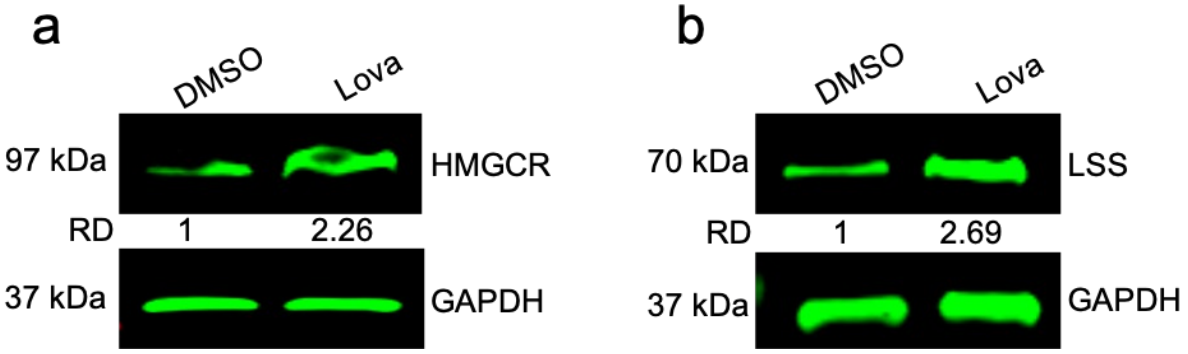

Supplementary Fig 1. Induction of the cholesterol biosynthesis proteins by lovastatin. (a,b) Induction of cholesterol biosynthesis proteins HMGCR (a) and LSS (b) by lovastatin, using western blot.

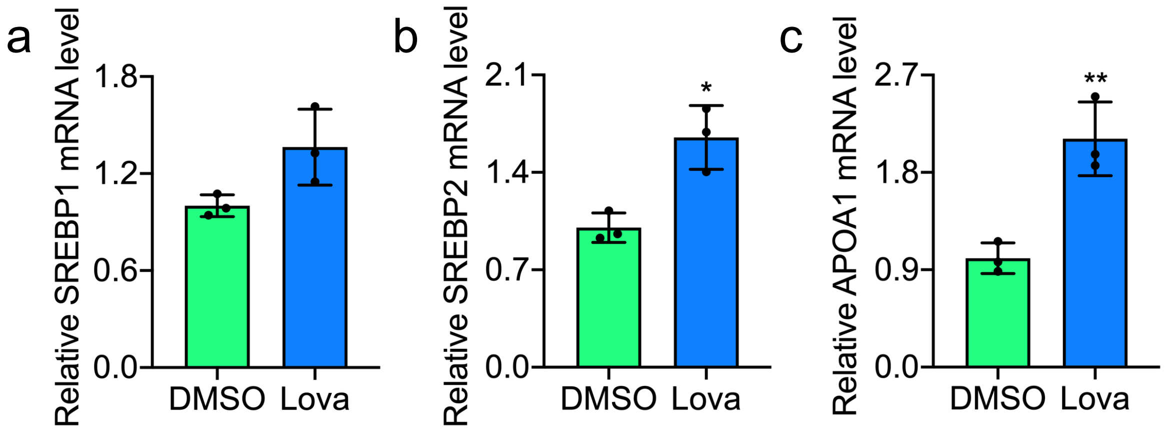

Supplementary Fig. 2. Effect of lovastatin on regulation of cholesterol biosynthesis genes. **(a-c)** HEL cells were treated with 20 μM of lovastatin and subjected for RT-PCR analysis of the SREBP1 **(a)**, SREBP2 **(b)** and APOA1 **(c)** genes.

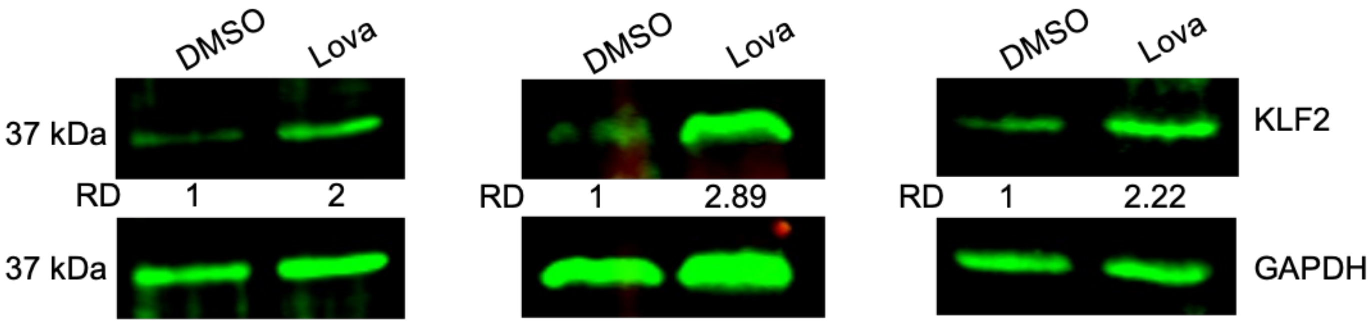

Supplementary Fig. 3. High expression of KLF2 in leukemia cells treated with lovastatin. The expression of KLF2 protein in the treated and untreated lovastatin leukemia spleens. GAPGH was used as loading control. Relative density (RD) measured using densitometer.

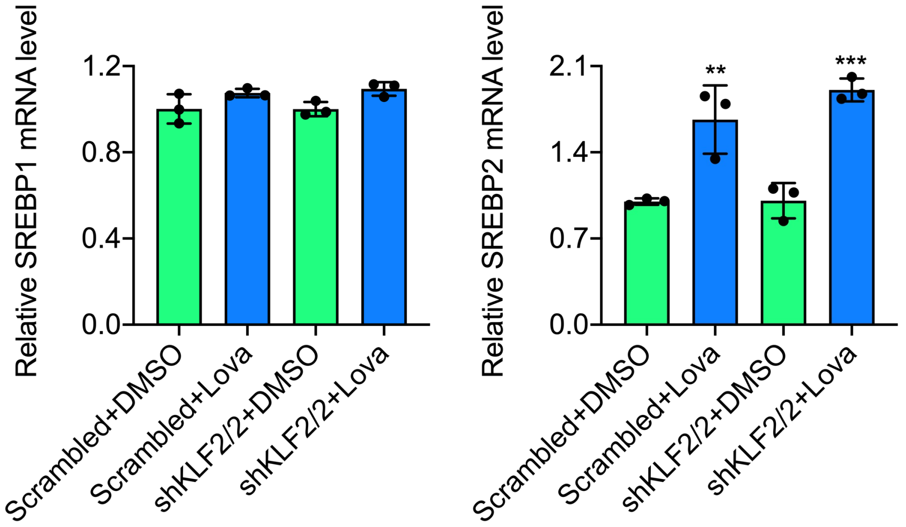

Supplementary Fig. 4. KLF2 knockdown effects the expression of SREBP2. Scrambled and shKLF2/2 cells were treated with lovastatin (20 μM) for 24 h and subjected for RT-qPCR for expression of the *SREBP1* and *SREBP2* genes, as indicated.
