# Supplementary material for: Lovastatin inhibits erythroleukemia progression through KLF2-mediated suppression of MAPK/ERK signaling
Source: BMC Cancer. 2023 Apr 4;23:306. doi: 10.1186/s12885-023-10742-4 (PMC10071686; doi:10.1186/s12885-023-10742-4)
Supplement: Supplementary file 3 — Supplementary Material 3 [file 12885_2023_10742_MOESM3_ESM.pdf]

Original/unprocessed western blot images

Fig. 1d

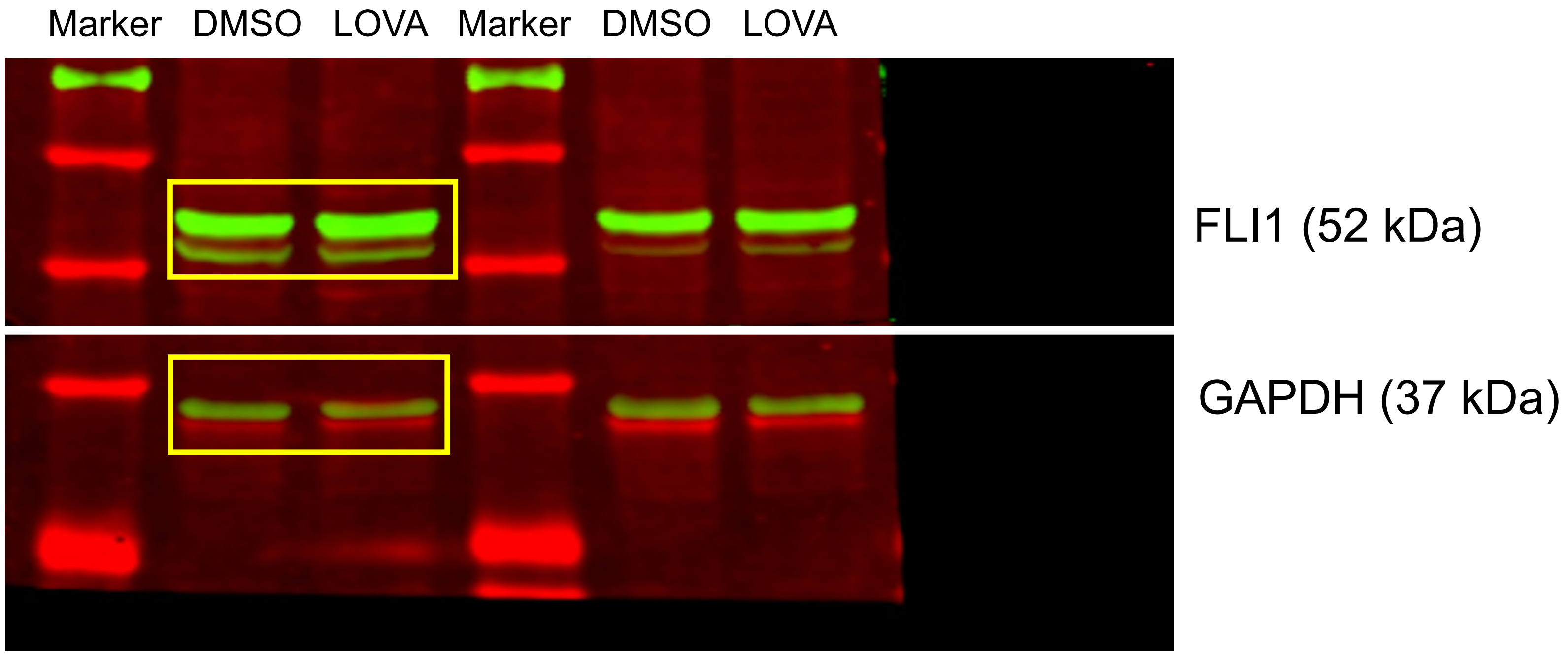

Supplementary Figure 5. The full-length blots of FLI1 and GAPDH. The same membranes were cut or/and hybridized with different antibodies according to the molecular weights.

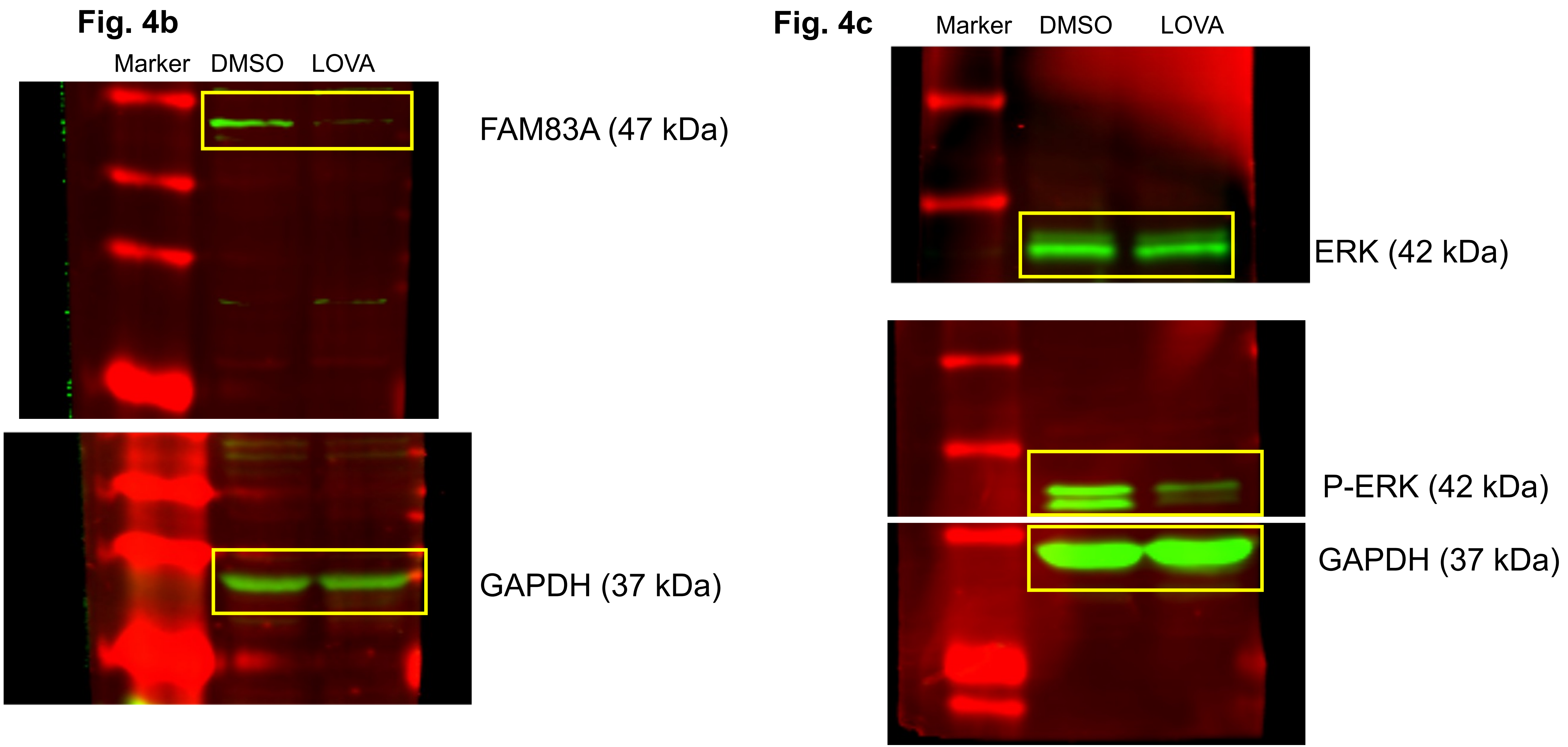

Supplementary Fig. 6. The full-length blots of FAM83A ERK, DDIT4, P-ERK and GAPDH. The same membranes were cut or/and hybridized with different antibodies according to the molecular weights.

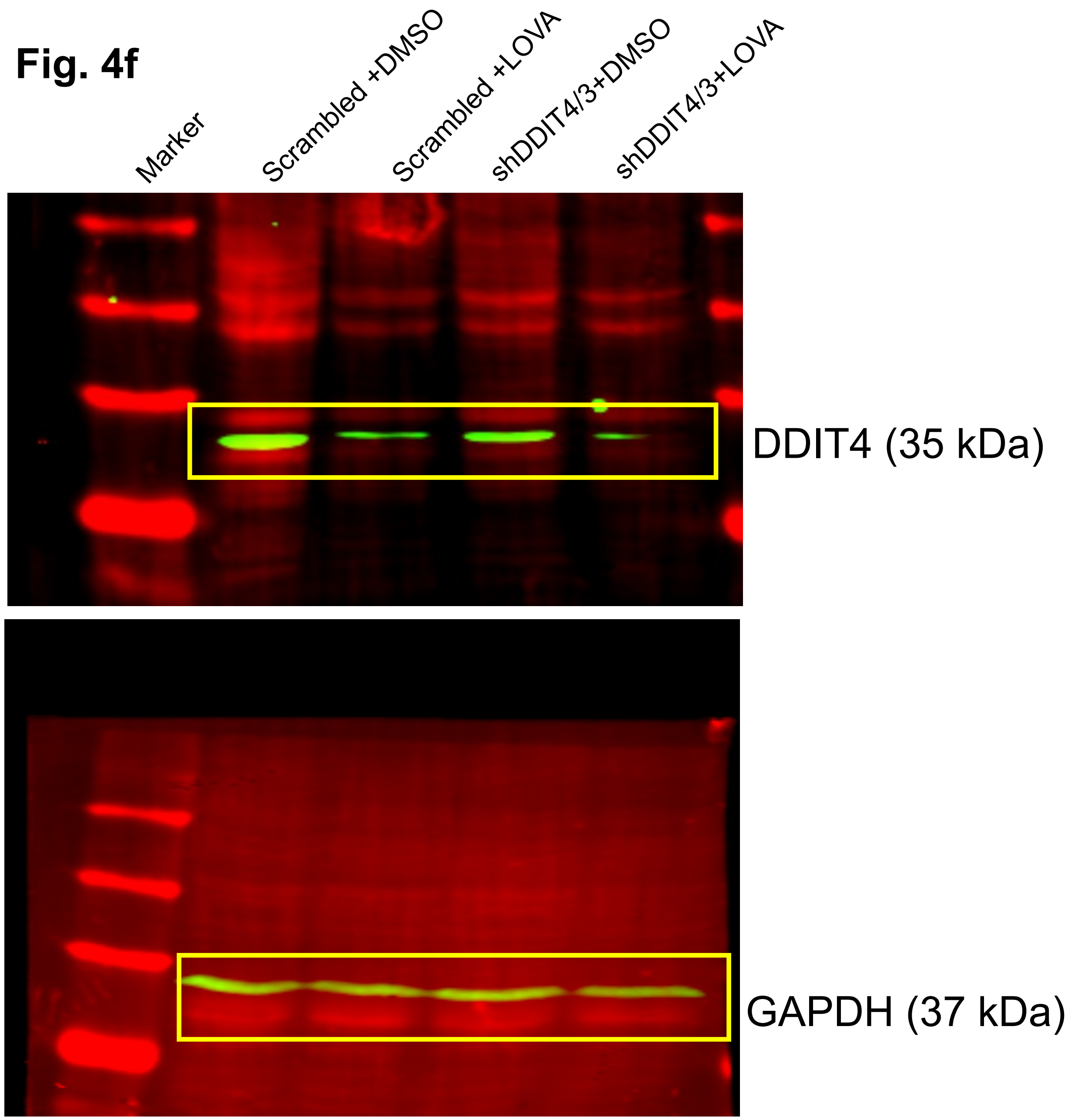

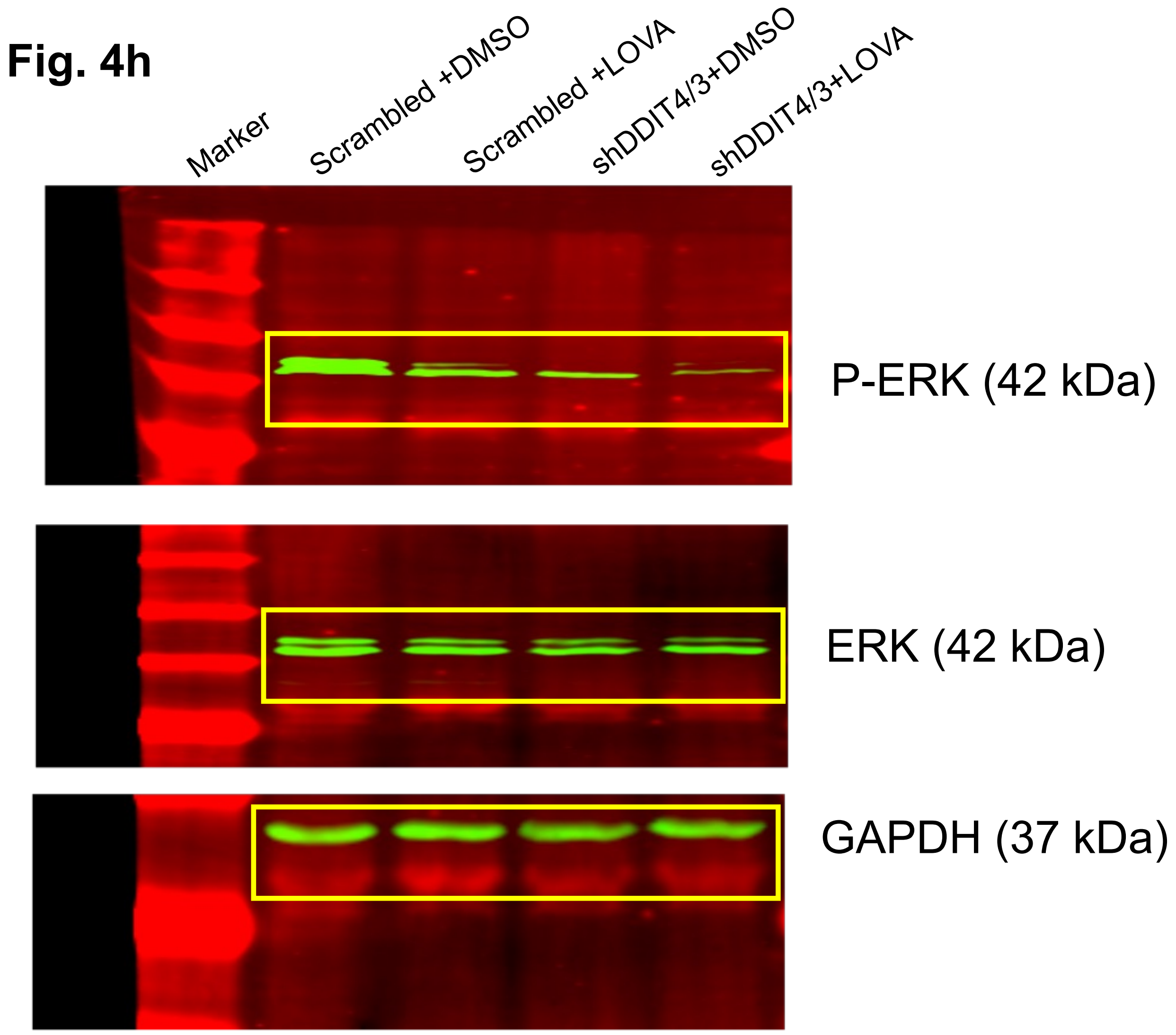

Supplementary Fig. 6. The full-length blots of FAM83A ERK, DDIT4, P-ERK and GAPDH. The same membranes were cut or/and hybridized with different antibodies according to the molecular weights.

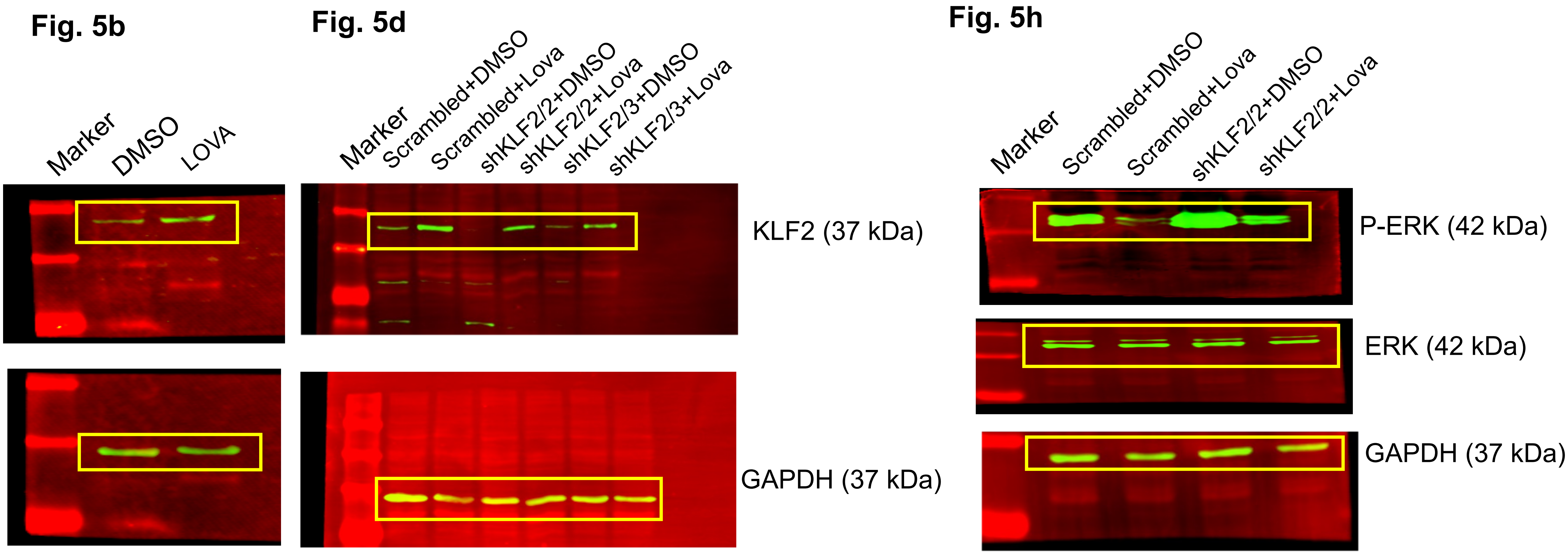

Supplementary Fig. 7. The full-length blots of KLF2, ERK, P-ERK and GAPDH. The same membranes were cut or/and hybridized with different antibodies according to the molecular weights.

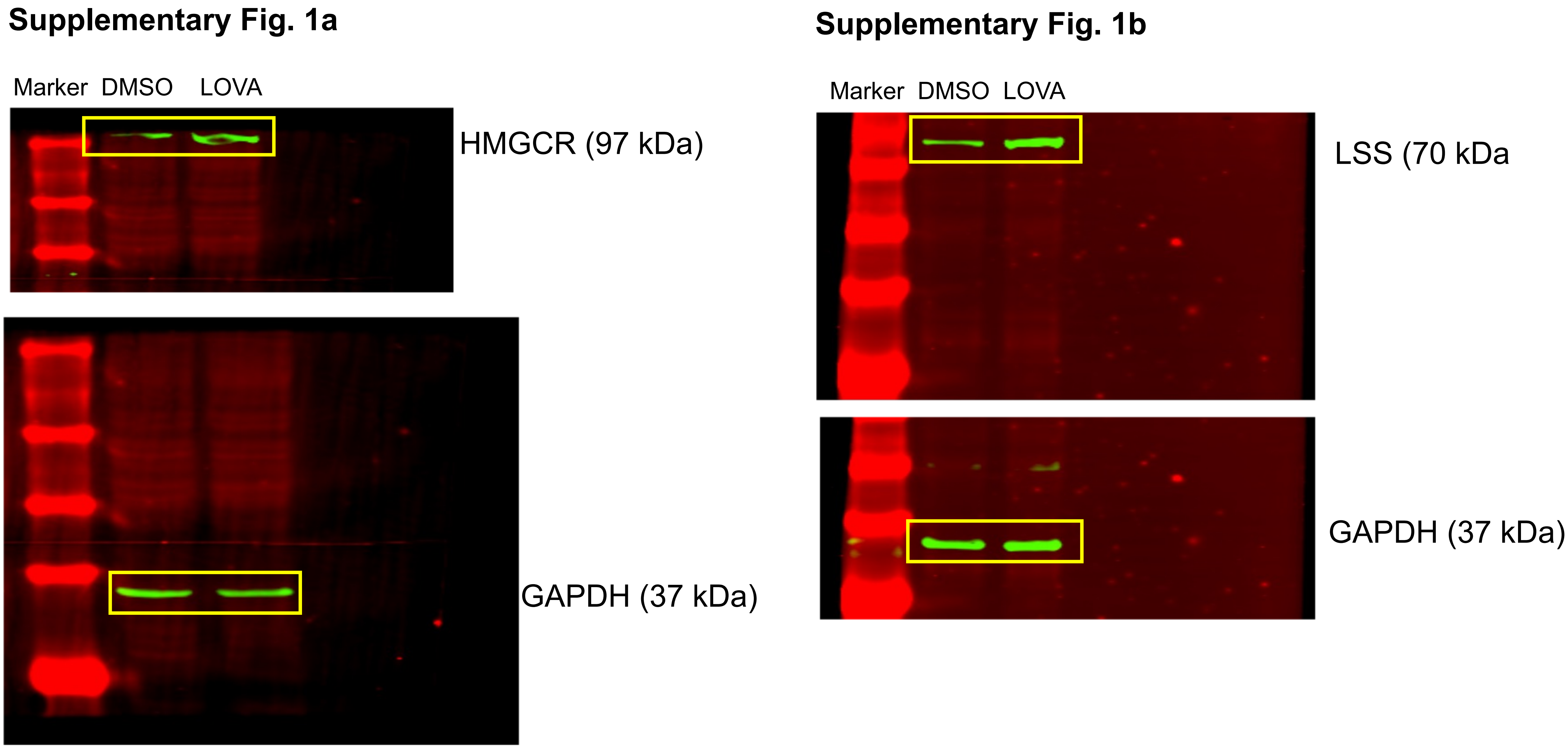

Supplementary Fig. 8. The full-length blots of HMGCR, LSS, and GAPDH. The same membranes were cut or/and hybridized with different antibodies according to the molecular weights.

Supplementary Fig. 3

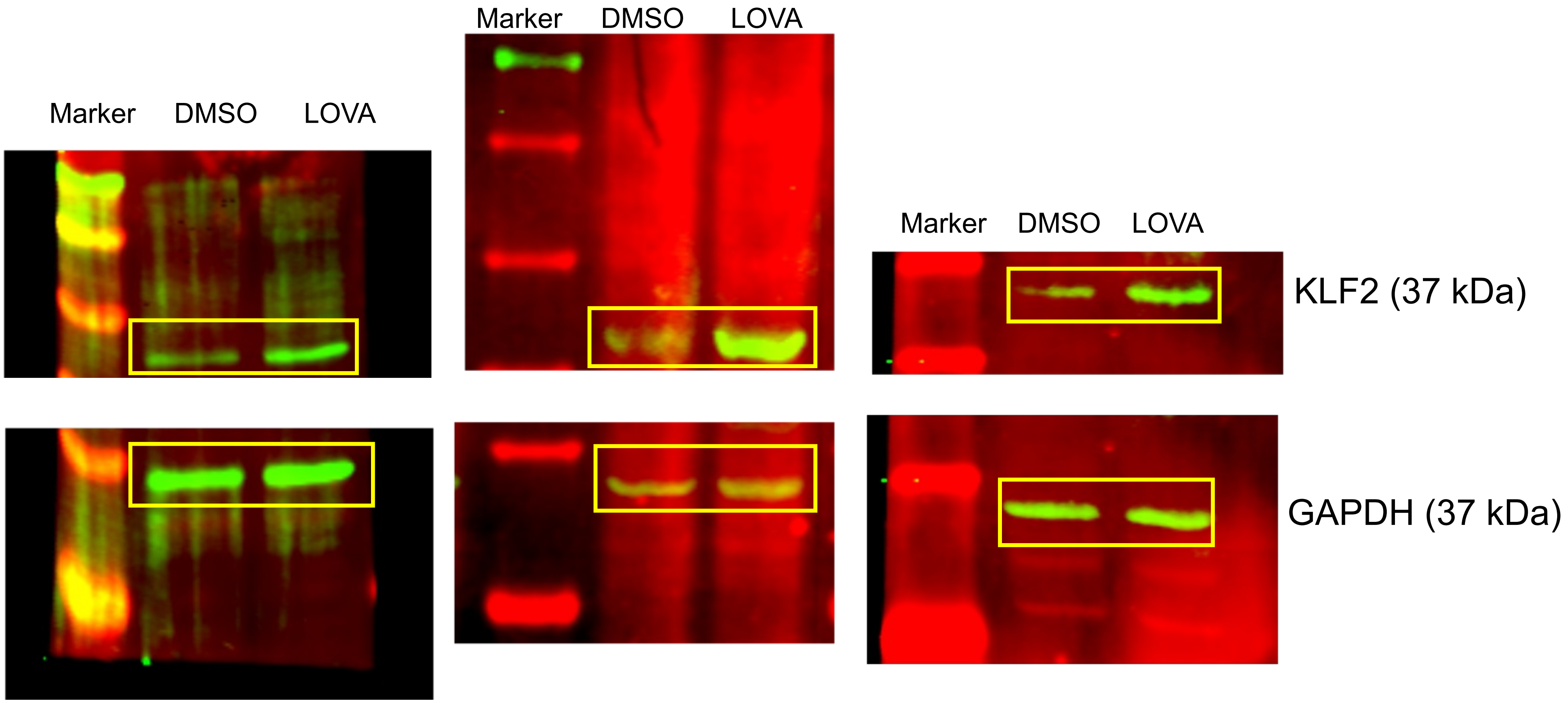

Supplementary Fig. 9. The full-length blots of KLF2 and GAPDH. The same membranes were cut or/and hybridized with different antibodies according to the molecular weights.
